# Two New Antiprotozoal Diterpenes From the Roots of *Acacia nilotica*

**DOI:** 10.3389/fchem.2021.624741

**Published:** 2021-04-21

**Authors:** John V. Anyam, Priscilla E. Daikwo, Marzuq A. Ungogo, Nwakaego E. Nweze, Ngozichukwuka P. Igoli, Alexander I. Gray, Harry P. De Koning, John O. Igoli

**Affiliations:** ^1^ Phytochemistry Research Group, Department of Chemistry, University of Agriculture, Makurdi, Nigeria; ^2^ Institute of Infection, Immunity and Inflammation, College of Medical, Veterinary and Life Sciences, University of Glasgow, Glasgow, United Kingdom; ^3^ Department of Veterinary Pharmacology and Toxicology, Ahmadu Bello University, Zaria, Nigeria; ^4^ Department of Veterinary Medicine, Faculty of Veterinary Medicine, University of Nigeria, Nsukka, Nigeria; ^5^ Centre for Food Technology and Research, Benue State University, Makurdi, Nigeria; ^6^ Strathclyde Institute of Pharmacy and Biomedical Science, University of Strathclyde, Glasgow, United Kingdom

**Keywords:** Nigeria, spectroscopy, *Acacia nilotica*, diterpenes, seco-oxocassanes, trypanosomiasis, *Leishmania*

## Abstract

The powdered roots of the medicinal plant *Acacia nilotica* were extracted with hexane and ethyl acetate, and the extracts were subjected to column chromatography for the isolation of potentially bioactive compounds and their screening against kinetoplastid pathogens. NMR and HREI mass spectrometric analyses identified two new diterpenes, characterized as 16, 19-dihydroxycassa-12-en-15-one (Sandynone, **1**) and (5S, 7R, 8R, 9R, 10S, 13Z, 17S)-7,8:7,17:16,17-triepoxy-7,8-seco-cassa-13-ene (niloticane B, **2**). The previously reported (5S,7R,8R,9R,10S) -(-)-7,8-seco-7, 8-oxacassa-13,15-diene-7,17-diol (**3**), (5S,7R,8R,9R,10S) -(-)-7,8-seco-7, 8-oxacassa-13,15-dien-7-ol-17-al (**4**), and (5S,7R,8R,9R,10S) -(-)-7,8-seco-7, 8-oxacassa-13,15-dien-7-ol (**5**) a, mixture of stigmasterol (**6a**) and sitosterol (**6b**), and lupeol (**7**) were also isolated. Several column fractions displayed significant activity against a panel of *Trypanosoma* and *Leishmania* spp., and from the most active fraction, compound **4** was isolated with high purity. The compound displayed high activity, particularly against *T. brucei*, *T. evansi*, and *L. mexicana* (0.88–11.7 µM) but only a modest effect against human embryonic kidney cells and no cross-resistance with the commonly used melaminophenyl arsenical and diamidine classes of trypanocides. The effect of compound **4** against *L. mexicana* promastigotes was irreversible after a 5-h exposure, leading to the sterilization of the culture between 24 and 48 h.

## Introduction

Parasitic kinetoplastid diseases, including trypanosomiasis and leishmaniasis, threaten millions of people in resource-poor countries around the world. *Trypanosoma* spp. and *Leishmania* spp., belonging to the family Trypanosomatida and the order Kinetoplastida, are among the most important agents of neglected tropical diseases (Butler, 2007; Vieira de Morais et al., 2015). These diseases occur mostly in the tropics where the humidity and high environmental temperatures favor both vector and parasite growth and attract insufficient resources (Patz et al., 2000).

African trypanosomiasis is endemic in 36 sub-Saharan African countries, including Nigeria, where there are tsetse flies that transmit the disease. While the number of new human African trypanosomiasis (HAT, or sleeping sickness) infections has significantly decreased in recent years, with only 977 cases recorded in 2018 (WHO, 2019b), African animal trypanosomiasis (AAT) still remains a major constraint to the use of livestock in the region (Geerts et al., 2001). About 50 million heads of cattle are exposed to AAT, and 35 million doses of trypanocides are used annually (Mattioli et al., 2004) in prevention and treatment. The direct and indirect losses of AAT are put at about US$ 4.5 billion (Geerts et al., 2001). Beyond Africa, surra and dourine, caused by *T. evansi* and *T. equiperdum*, respectively, affect millions of high-value animals in Asia, Europe, Australia, and South America (Brun et al., 1998; Desquesnes et al., 2013).

Leishmaniasis generally affects the poorest of the poor and is associated with malnutrition, population displacement, poor housing, a weak immune system, and a lack of financial resources (WHO, 2019a). The disease manifests as visceral, cutaneous, and mucocutaneous infections. It is endemic in 98 countries, predominantly in Latin America, South and Central Asia, and parts of Africa (Alvar et al., 2012) where approximately 350 million people are at risk of contracting the infection. An estimated 700,000 to 1 million new cases and some 26,000 to 65,000 deaths occur annually (WHO, 2019a).

Nigerian medicinal plants are a rich source of natural compounds with potent antiprotozoal activity (Ungogo et al., 2020). *Acacia nilotica* Linn. (Mimosaceae) is a common medicinal plant found in subtropical and tropical Africa from Nigeria to Egypt and South Africa and other parts of the world (Chowdhury et al., 1983; Van Wyk et al., 1997 Roozbeh and Darvish, 2016). The plant is a small- to medium-size tree (Boulos, 2000), 7–13 m tall, with a stem diameter of 20–30 cm. The bark is dark brown to black. It has bright yellow flowers with bipinnate leaves. The plant is attractive to a wide range of pests, diseases, and wild animals (Sheik, 1989). There are several ethnobotanical uses of the plant, including treatment of abdominal pain, diarrhea, dysentery, and genital and urinary tract infections, and as an expectorant (Boulos, 1983)*.* It possesses antimicrobial, antiplasmodial, antihypertensive, and antioxidant activities (Ali et al., 2012). Niloticane, a cassane diterpene (Eldeen et al., 2010), and umbelliferone, a coumarin (Singh et al., 2010), have been isolated from the plant, as well as some flavonoids and phenolic compounds (Saleem, 2011). Other *Acacia* species have yielded seco-oxacassanes and unusual diterpenoids such as schaffnerine, isolated from *Acacia schaffneri* (Manríquez-Torres et al., 2011; Manríquez-Torres et al., 2013). In the present study, we have carried out further phytochemical studies on *Acacia nilotica* and hereby report the isolation and characterization of two novel diterpenes from the roots.

Most of the drugs available for the treatment of trypanosomiasis and leishmaniasis are outdated and associated with toxic side effects, prolonged duration of treatment, and resistance (Anene et al., 2001; WHO, 2010; De Koning, 2020). Therefore, there is an urgent need for new drugs for the treatment of trypanosomiasis and leishmaniasis, and the active ingredients of traditionally used medicinal plants are a prime source of unrelated, new compounds. Hence, the compounds and mixtures obtained from *A. nilotica* were also investigated for antitrypanosomal and antileishmanial activities as well as for toxicity against human cell lines *in vitro*. Activity-guided fractionation yielded one pure compound with activity against several *Trypanosoma* species and against *Leishmania mexicana* below 0.5 μg/ml. The effect on *Leishmania mexicana* promastigotes was irreversible within 5 h and fatal after 24 h.

## Materials and Methods

### General Experimental Procedures

Column chromatography was carried out using silica gel 60 (0.040–0.063 mm) (230–400 mesh ASTM). Thin-layer chromatography (TLC) was performed on precoated aluminum sheets coated with silica gel F250 (Merck, Germany). Nuclear magnetic resonance (NMR) experiments were carried out on a Bruker AVIII (500 MHz) spectrophotometer using CDCl_3_ as the solvent and TMS as the internal standard. Mass spectral data were acquired on a JEOL MStation JMS-700 mass spectrometer.

#### Plant Material

Roots of *Acacia nilotica* were collected from trees growing on the campus of the University of Agriculture, Makurdi. The plant was authenticated at the Department of Forestry and Wildlife of the university and a voucher specimen deposited at their herbarium.

#### Isolation of Compounds

Dried roots of the plant were ground to powder (250 g) and extracted with hexane and ethyl acetate. The extracts were combined (based on similarity on TLC) and subjected to column chromatography using silica gel in a glass column. The column was packed wet in a hexane: ethyl acetate (95:5) mixture and eluted with ethyl acetate in hexane gradient starting with 5% ethyl acetate in hexane and increasing the amount of ethyl acetate by 5% until 100% ethyl acetate collecting 10-ml vials to obtain 186 fractions. The fractions were examined by TLC, and similar ones were combined and allowed to dry in a fume hood to obtain a mixture of compounds **1** and **3** (fractions 76–79), mixture of compounds **2** and **5** (fractions 18–19), compound **4** (fractions 64–67), a mixture of compounds **6a** and **6b** (fractions 59–60), and compound **7** (fractions 25–28) as white crystalline solids. The compounds were analyzed by NMR (1D and 2D) spectroscopy and mass spectrometry.

### Determination of Antiprotozoal and Cytotoxic Activity

#### Parasites, Mammalian Cells, and Culture Conditions

Two strains of *Trypanosoma brucei brucei* bloodstream form (BSF) were used in this study: 1) wild-type (WT) *T. b. brucei* strain Lister 427 (De Koning et al., 2000) and 2) a multidrug resistant strain, B48, which was derived from a TbAT1-KO strain (Matovu et al., 2003) after increasing *in vitro* exposure to pentamidine and lacks both the TbAT1/P2 transporter and the high-affinity pentamidine transporter (HAPT1) (Bridges et al., 2007). The two *T. b. brucei* strains and drug-sensitive (WT) strains of *T. evansi* and *T. equiperdum* were used throughout as bloodstream trypomastigotes and cultured in standard Hirumi’s modified Iscove’s medium 9 (HMI9), supplemented with 10% heat-inactivated fetal bovine serum (FBS), 14 μL/L β-mercaptoethanol, and 3.0 g/L sodium hydrogen carbonate (pH 7.4). The parasites were cultured in vented flasks at 37°C in 5% CO_2_ atmosphere and were passaged every 3 days (Rodenko et al., 2015). The bloodstream forms of *T. congolense* savannah-type strain IL3000 and *T. congolense* strain 6C3 [diminazene-resistant (Alenezi et al., 2020)] were cultured, as described by Coustou et al. (2010). *Leishmania mexicana* promastigotes (MNYC/BZ/62/M379 strain) were grown in hemoflagellate modified minimal essential medium (HOMEM) (Gibco®, Life technologies, Ghent, Belgium) (pH 7.4) supplemented with 10% heat-inactivated FBS at 27°C.

Human embryonic kidney (HEK) cells were cultured in Dulbecco’s modified Eagle’s medium (DMEM; Sigma D-5671) supplemented with 10% heat-inactivated FBS, 10 ml/L penicillin/streptomycin (Gibco 15140-122), and 10 ml/L of 200 mM glutamine (Gibco 25030-024). The cells were maintained at 37°C in 5% CO_2_ atmosphere.

#### Test Compounds/Fractions

All compounds and mixtures were dissolved in DMSO at 10 mg/ml, and the stock solutions were stored at −20°C.

#### *In vitro* Drug Sensitivity Assay Using Resazurin (alamarBlue) in Bloodstream Forms of *T. b. brucei*, *T. equiperdum*, *T. evansi*, and *T. Congolense*

The susceptibilities of bloodstream formtrypanosomes to the compounds and mixtures were determined using resazurin (alamarBlue)-based assay, as described previously (Nvau et al., 2020). In brief, serial double dilutions of the test compounds were prepared in cell-specific medium in 96-well plates (200 μg/ml top concentration: 23 dilutions over 2 rows, last well drug-free control). This is followed by the addition of 100 µL of parasite suspension in the appropriate medium, to each well of the 96-well plate, adjusted to the desired cell density. For *T. brucei* s427, *T. brucei* B48, and *T. equiperdum*, a seeding density of 2 × 10^4^ cells/well was used, whereas cell densities of 4 × 10^4^ and 5 × 10^4^ were used for *T. evansi* and the *T. congolense* strains (IL3000 and 6C3), respectively. Trypanosome cultures with the test drugs were incubated for 48 h, followed by the addition of 20 µL of filter-sterilized 125 μg/ml resazurin sodium salt in phosphate-buffered saline (PBS). This was followed by a further 24 h of incubation. Standard drugs including diminazene aceturate and suramin were used as positive control as appropriate for the species. Fluorescence was measured in 96-well plates with a FLUOstar Optima (BMG Labtech, Durham, NC, United States) at wavelengths of 544 nm for excitation and 590 nm for emission. EC_50_ values were calculated by nonlinear regression using an equation for a sigmoidal dose–response curve with variable slope (GraphPad 7.0, GraphPad Software Inc., San Diego, CA, United States).

#### Drug Sensitivity Using alamarBlue in *L. mexicana* Promastigotes

Drug sensitivity assay in *L. mexicana* was carried out using a similar method as described above. However, a seeding density of 2 × 10^5^ cells/well was used for this species. The plate containing the cells and drug dilutions was incubated for a period of 72 h at 27°C, followed by the addition of 20 µL 125 μg/ml resazurin and a further 48 h of incubation. Pentamidine was used as a control drug. Fluorescence was measured as above.

#### Assessment of Cytotoxicity of Test Compounds on Human Embryonic Kidney (HEK) 293T Cells

HEK cells were harvested at 80–85% confluence using 0.25% Trypsin–EDTA solution (Sigma T-4049). The cells were washed by centrifugation at 1200 rpm for 10 min and reconstituted in fresh medium at 3 × 10^5^ cells/ml. Then, 100 µL of the cell suspension was distributed to each well of a 96-well plate and incubated for 24 h to allow the cells to adhere to the bottom of the wells. Doubling serial dilutions of the test compounds and control drug were prepared in a separate 96-well plate, across 1 row (11 dilutions plus no-drug control). And then, 100 µL of each dilution was transferred to respective wells of the plate containing the cells, and the plate was incubated for another 30 h. This was followed by the addition of 10 µL 125 μg/ml resazurin sodium salt to each well, and a further incubation was done for 24 h. The plates were read and EC_50_ determined as above. The selectivity index (SI) was also calculated for each compound/mixture as the ratio of the EC_50_ in HEK cells to the EC_50_ in a parasite species.

#### Determination of the Effect of HEAN 19b on *L. mexicana* Growth

*L. mexicana* cultures were set at 10^6^ cells/ml in a 24-well plate with or without varying concentrations of HEAN-19b and pentamidine. Depending on intended duration of exposure, cells were centrifuged at 5 h and 48 h and resuspended in either fresh medium or medium containing the drug. Cells were counted manually using a hemocytometer, and a growth curve was plotted using cell density at each time recorded.

## Results and Discussion

### Structure Elucidation

**FIGURE 1:**
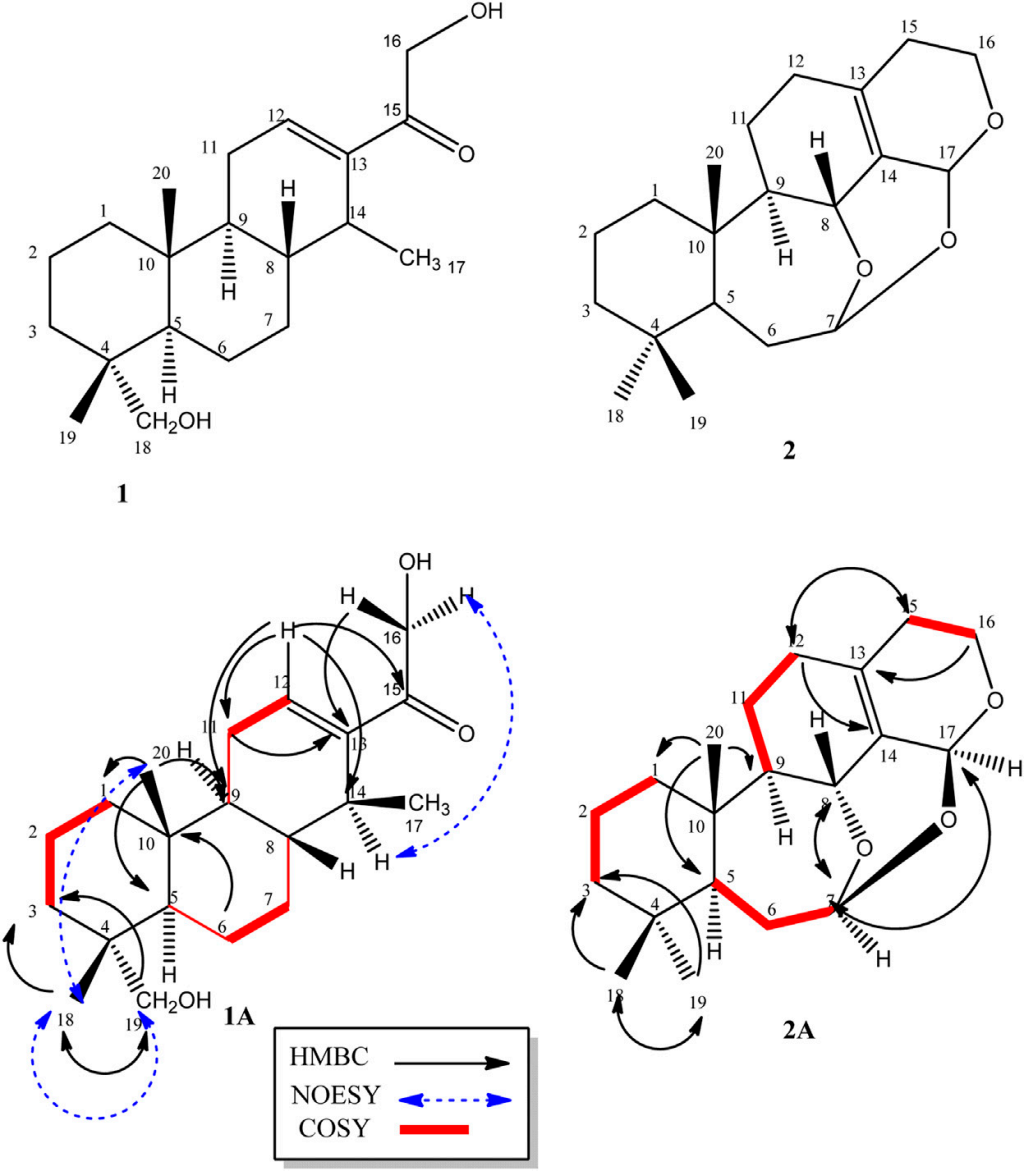
Structure of compounds **1** and 2, including their selected COSY and HMBC correlations (1A and 1B).

Compound **1**, a white crystalline solid, was obtained as the minor component of combined fractions 76-79. The molecular formula C_20_H_32_O_3_ was derived from its high resolution mass spectrum (Supplementary Material 1), which yielded an [M-H]^−^ ion at *m*/*z* = 319.2229 (calculated 319.2273 for C_20_H_31_O_3_). Its proton spectrum (Supplementary Material 2 and Table 1) showed an olefinic proton at *δ*_H_ 6.74 ppm (1H, t, *J* = 4.0 Hz, H-12) and two sets of oxymethylene protons at 4.44 (1H, d, *J* = 17.6 Hz, H-16), 4.56 (1H, d, *J* = 17.6 Hz, H-16), 3.12 (1H, d, *J* = 10.8 Hz, H-19), and 3.42 (1H, d, *J* = 10.8 Hz, H-19). It also displayed signals for three methyl protons made up of a methyl doublet at 0.86 (3H, d, *J* = 6.2 Hz, H-17) and two singlets at 0.80 (3H, s, H-18) and 0.88 (3H, s, H-20). The rest of the signals were for three methine (including a quartet at 2.70 (1H, q, *J* = 6.2 Hz, H-14) and six pairs of methylene protons. Its ^13^C spectrum (Supplementary Material 3) gave signals for 20 carbon atoms including one ketone carbonyl at *δ*_C_ 198.0 and two hydroxyl bearing carbons at 72.1 and 64.4 ppm. There were also two olefinic carbons, one proton bearing at 141.0 and the other a quaternary at 142.0. The rest of the signals were for three methyls, six methylenes, four methines, and two quaternary carbons. Using correlations in its 2D NMR spectra (Supplementary Material 4–6), the structure (Figure 1) was deduced as follows: correlations from the olefinic proton at 6.74 ppm to the carbonyl carbon at 198.0 (C-15) indicated it was three bonds away from it. Other correlations from the olefinic proton are to C-9 and C-14 and to this was attached the proton quartet; hence, the methyl doublet C-17 must be attached at C-14. This was confirmed by correlations from H-14 to C-12, C-15, and C-17. Others were ^2^ *J* long-range correlations from H-16 to C-15 and H-18 to C-19, thus confirming by HMBC and HSQC, H-16, C-16 and H-19, C-19 to be -CH_2_OH (hydroxymethylene groups). The absence of germinal methyl groups usually at C-4, further confirmed C-19 to be a hydroxymethylene carbon and C-18 a methyl carbon. Therefore, correlations from H-19 and H-18 were used to identify C-3, C-4, and C-5 and from H-5 to identify C-1, C-7, C-10, and C-20 and confirm C-9. The compound was identified as 16, 19-dihydroxycassa-12-en-15-one and given the trivial name Sandynone **1**, and its chemical shifts compared very well with similar compounds (Mendoza et al., 2003). The relative stereochemistry was determined using its NOESY (Supplementary Material 7) and is shown in Figure 1.

**TABLE 1:** ^1^H NMR data for compounds **1** (at 500 MHz) and **2** (at 400 MHz) in CDCl_3_.

	Compound **1**			Compound **2**		Literature report (compd **2**)^a^
**Position**	^**1**^ **H (δ in ppm; mult, *J* in Hz)**	^**13**^ **C (mult)**	^**1**^ **H (δ in ppm; mult, *J* in Hz)**	^**13**^ **C (mult)**	^**1**^ **H (δ in ppm)**	^**13**^ **C**
1	0.97, 1.62	38.9 (CH_2_)	0.98, 1.97	40.9 (CH_2_)	0.94, 1.94	40.7
2	0.82 (m)	18.0 (CH)	1.44, 1.51	18.9 (CH_2_)	1.40, 1.47	18.7
3	1.41, 1.30	35.4 (CH_2_)	1.16, 1.42	41.8 (CH_2_)	1.14, 1.43	41.6
4	—	37.7 (C)	—	34.5 (C)	—	34.3
5	1.22	48.4 (CH)	1.23 (br d)	47.8 (CH)	1.28	47.6
6	1.57 (m)	21.3 (CH_2_)	1.83, 1.61	31.6 (CH_2_)	1.65, 1.87	31.4
7	1.67 (m)	30.5 (CH_2_)	5.21 (dd, 10.4,5.2)	95.1 (CH)	5.26	94.9
8	1.51	34.9 (CH)	4.47 (d, 8.9)	67.4 (CH)	4.52	67.2
9	1.38 (d, 3.7)	43.9 (CH)	1.23 (d, 4.0)	56.6 (CH)	1.21	56.4
10	—	36.7 (C)	—	39.2 (C)	—	39.0
11	2.23 (d,4.3), 2.08	26.1 (CH_2_)	1.74, 1.21 (d, 4.0)	21.4 (CH_2_)	1.76, 1.30	21.2
12	6.74 (t, 4.0)	141.0 (CH)	1.78 (m), 2.04 (d, 2.1)	32.0 (CH_2_)	2.09, 1.83	31.8
13	—	142.0 (C)	—	135.9 (C)	—	135.8
14	2.70 (q, 6.2)	31.4 (CH)	—	129.9 (C)	—	129.6
15	—	198.0 (C)	2.31 (d, 7.1), 1.64 (d. 5.3)	30.4 (CH_2_)	2.37, 1.30	30.2
16	4.44 (d, 17.6), 4.56 (d, 17.6)	64.4 (CH_2_)	3.84 (td, 11.4, 3.8), 3.69 (dd, 11.0, 5.7)	56.4 (CH_2_)	3.66, 3.82	56.2
17	0.86 (d, 6.2)	15.1 (CH_3_)	5.72 (br s)	88.6 (CH)	5.70	88.4
18	3.12, 3.42	72.1 (CH_2_)	0.89 (s)	33.2 (CH_3_)	0.87	33.0
19	0.80	18.0 (CH_3_)	0.87 (s)	22.5 (CH_3_)	0.85	22.3
20	0.88	22.5 (CH_3_)	0.90 (s)	15.7 (CH_3_)	0.88	15.5

^a^ Manríquez-Torres et al. (2013).

Compound **2** was also isolated as a white crystalline solid. The molecular formula was established based on its [M]^+^ ion in its HR-EIMS spectrum (Supplementary Material 12) at *m*/*z* = 318.2195 (calculated 318.2195 for C_20_H_30_O_3_). The ^1^H NMR spectrum (Supplementary Material 13 and Table 1) showed two acetal protons at *δ*_H_ 5.70 (br s, H-17) and 5.26 (dd, *J* = 10.4, 5.2 Hz, H-7), a methine geminal to oxygen at δH 4.52 (br d, *J* = 8.0 Hz, H-8), and two methylene protons H-16 geminal to oxygen at *δ*_H_ 3.77 (dt, *J* = 11.4, 3.9 H-16 β) and 3.61 (ddd, *J* = 11.4, 6.6, 1.2, H-16α) coupled with the H-15 methylene protons at *δ*_H_ 2.31 and 1.64 ppm. There were three methyl groups observed at *δ*_H_ 0.83 (Me-20), 0.80 (Me-19), and 0.83 (Me-18). In the ^13^C DEPT-135 spectrum (Supplementary Material 14), 20 signals were observed including two quaternary olefinic carbons at *δ*_C_ 135.9 (C-13) and 129.9 (C-14), two acetal carbons at *δ*_C_ 95.1 (C-7) and 88.6 (C-17), one oxymethylene carbon at 56.4 (C-16), and an oxymethine carbon at 67.4 (C-8). The rest of the signals were for two methine (C-5, C-9), seven methylene, three methyl, and two quaternary carbons. The structure was confirmed using its 2D NMR spectra (Supplementary Material 15–16) as follows: long-range correlations from the acetal proton H-7 identified carbon C-5, C-8, and C-17, while from the second acetal proton H-17, carbons C-7, C-8, C-13, C-14, and C-16. Similarly, long-range correlations from H-18 and H-19 indicated they were germinal and identified C-3, C-4, and C-5, and H-20 also confirmed C-5 as well identifying C-1, C-9, and C-10. The HSQC spectrum confirmed the proton bearing carbons in the compound and their attached protons, while the COSY spectrum confirmed neighbouring protons. Compared to the macrocyclic dimer isolated from *Acacia schaffneri* (Manríquez-Torres, et al., 2013), their chemical shifts were identical (Table 1); however, the exact mass obtained was for a monomer with the acetal link between C-7 and C-17. There was no mass higher than the molecular ion in its HR-EIMS spectrum and no mass fragment which could suggest the compound being dimeric. Hence the presence of a dimer or the compound existing as a dimeric molecule could not be confirmed, and the compound was therefore identified as the seco-oxacassane, (5S, 7R, 8R, 9R, 10S, 13Z, 17S)-7,8:7,17:16,17-triepoxy-7,8-seco-cassa-13-ene (niloticane B, **2**) and confirmed by literature reports (Manríquez-Torres, et al., 2013).

**FIGURE 2:**
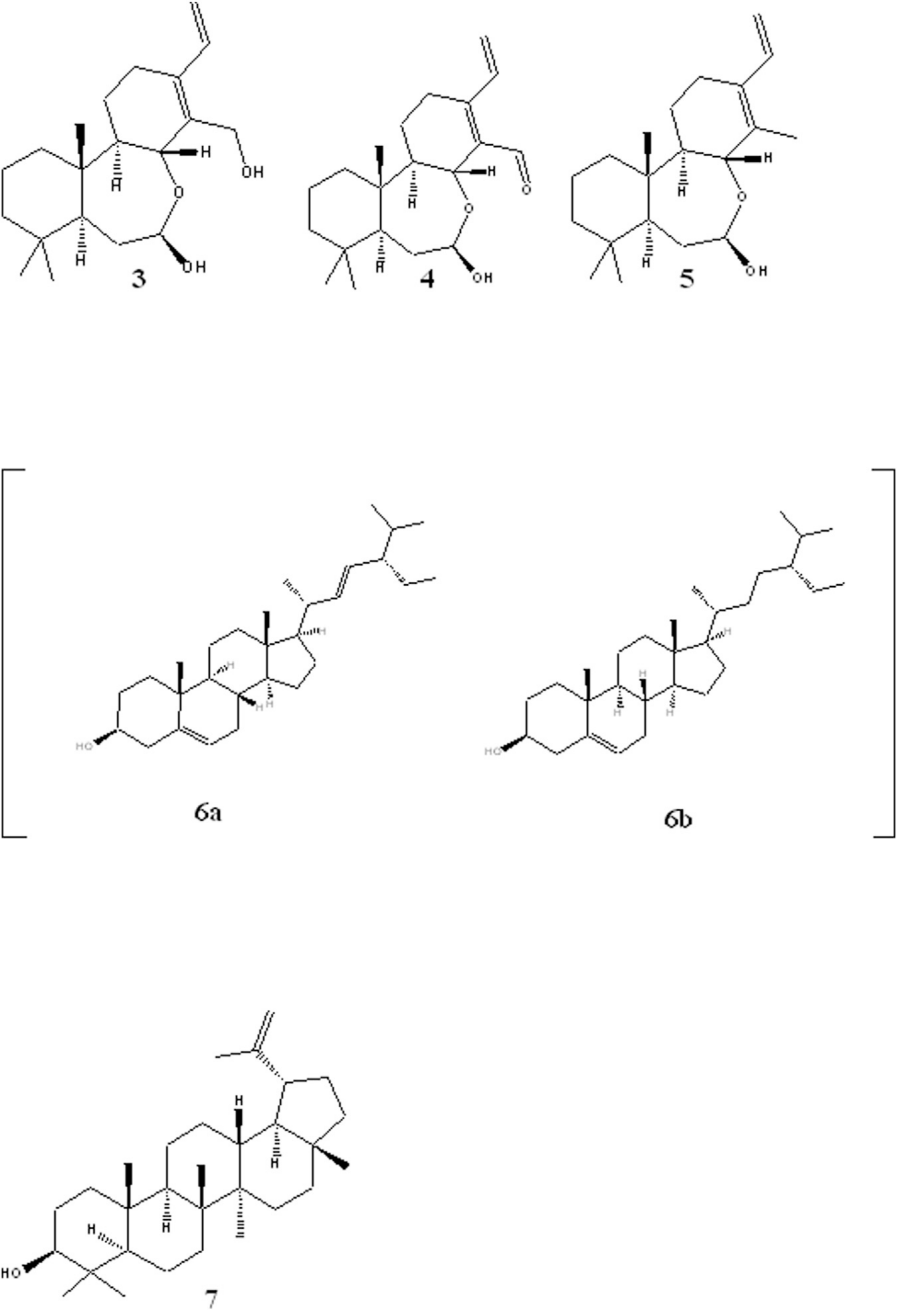
Structures of other isolated compounds: oxacassadienes (**3**, **4** and **5**); stigmasterol **6a** and β-sitosterol **6b** and lupeol **7**.

The rest of the compounds (Figure 2) were identified based on their NMR spectra and comparison with literature reports. Compound **3** was identified as (5S,7R,8R,9R,10S) -(-)-7,8-*seco*-7, 8-oxacassa-13,15-diene-7,17-diol, compound **4** as 5S,7R,8R,9R,10S) -(-)-7,8-*seco*-7, 8-oxacassa-13,15-dien-7-ol-17-al and compound **5** as (5S,7R,8R,9R,10S) -(-)-7,8-seco-7, 8-oxacassa-13,15-dien-7-ol (Manríquez-Torres et al., 2011). While compounds **6a** and **6b** were identified as a mixture of sitosterol and stigmasterol and compound **7** as lupeol.

### Antiprotozoal and Cytotoxic Activity of Fractions and Isolated Compounds

Two compound mixtures and a pure compound were tested for activity against bloodstream forms of four species of *Trypanosoma* and *L. mexicana* promastigotes using resazurin-based drug sensitivity assay. This assay is cheap and allows for efficient and reproducible screening of compounds for activity against cultured cells and is therefore commonly employed in parasitology (Räz et al., 1997; Gould et al., 2008). In addition, the compound and mixtures were investigated for toxicity in HEK cells and for cross-resistance to the commonly used melaminophenyl arsenical and diamidine classes of trypanocides (Bridges et al., 2007; Giordani et al., 2019). PAN-76, a mixture of compounds **1** (80%) and **3** (20%), showed promising antitrypanosomal activity with lowest EC_50_ of 4.27 ± 0.06 μg/ml against *T. evansi* (Table 2). Another mixture, HEAN-18, containing compounds **2** (70%) and **5** (30%), had only modest to poor activity against all the *Trypanosoma* and *Leishmania* species assayed. However, crude extract HEAN-1 displayed good activity against all the kinetoplastid species, except *T. congolense* (EC_50_ ≤ 10 μg/ml), and was thus selected for further purification, yielding compound **4**. This compound showed very potent antiprotozoal activity with EC_50_ as low as 0.45 ± 0.02 μg/ml, 0.33 ± 0.05 μg/ml, and 0.28 ± 0.05 μg/ml against *T. b. brucei*, T. *evansi*, and *L. mexicana*, respectively.

**TABLE 2:** EC_50_ of two mixtures and compound **4** against *Trypanosoma* and *Leishmania* species (*n* = 3).

**Compound/mixture**	***T. brucei* s427**	***T. congolense* IL3000 WT**	***T. equiperdum***	***T. evansi***	***L. mexicana***
PAN-76 compounds 1 and 3 (µg/ml)	10.1 ± 1.0	44.1 ± 3.2	7.2 ± 0.6	4.3 ± 0.1	34.1 ± 11.2
HEAN-18 compounds 2 and 5 (µg/ml)	25.9 ± 2.6	194.8 ± 98.9	17.2 ± 2.6	ND	51.9 ± 3.8
HEAN 1 crude extract containing compound 4 (µg/ml)	5.7 ± 0.1	35.8 ± 4.3	7.3 ± 1.7	5.4 ± 0.1	10.1 ± 0.6
HEAN 19b compound 4 (µg/ml)	0.45 ± 0.02 (1.41 µM)	3.72 ± 0.54 (11.7 µM)	1.39 ± 0.27 (4.36 µM)	0.33 ± 0.05 (1.04 µM)	0.28 ± 0.05 (0.88 µM)
Diminazene (µM)	0.0878 ± 0.0355	0.228 ± 0.0446	0.0382 ± 0.0050	0.0438 ± 0.0005	ND
Suramin (µM)	0.0189 ± 0.0004	8.74 ± 1.634	0.021 ± 0.006	ND	ND
Pentamidine (µM)	ND	ND	ND	ND	0.786 ± 0.022

ND, not done.

However, all of the mixtures and, to a lesser extent, compound **4**, exhibited only moderate to low activity against *T. congolense* IL3000, suggesting poor prospects for development as agents in the treatment of AAT, where the infecting species is usually not known, although applications outside the African tsetse belt (*T. evansi* and *T. equiperdum*) look more promising. It is a very common occurrence for drugs and test compounds to vary in activity between *T. congolense* and the species in the *T. brucei* group of species, making drug development for AAT even more challenging as drug candidates need to be active against three different species, that is, *T. b. brucei*, *T. congolense*, and *T. vivax*. In addition, while PAN-76 and HEAN-18, containing various amounts of compounds **1**, **2**, **3**, and **5**, demonstrated at best modest antileishmanial activity, whereas the purified compound **4** displayed an EC_50_ of 0.28 ± 0.05 μg/ml (Table 2).

Due to the enormous challenge drug resistance poses to the control of trypanosomiasis and leishmaniasis (De Koning, 2017), it is important that test compounds are tested for the prospect of cross-resistance to existing drugs. Neither of the three mixtures nor compound **4** was cross-resistant to diminazene and pentamidine as there were no significant differences (*p* > 0.05) between EC_50_ in the *T. brucei* WT and multidrug-resistant B48 strain, and between the *T. congolense* WT and the diminazene-resistant 6C3 strain (Table 3). This also means that for the *brucei* group species, there is no cross-resistance with melaminophenyl arsenical compounds such as melarsoprol and cymelarsan, which, like pentamidine, rely on the P2 and HAPT1 transporters for their trypanocidal activities (Bridges et al., 2007; Munday et al., 2015). In all cases for the mixtures and compound 4, the resistance factor (RF), which is the ratio of the EC_50_ in a drug-resistant strain over the EC_50_ of the WT control, was ≤1.4.

**TABLE 3:** Cross-resistance of two mixtures and compound 4 with existing drugs.

**Compound/mixture**	***T. brucei* B48**		***T. congolense* 6C3**	
	**RF**	***p* value**	**RF**	***p* value**
PAN-76 compounds 1 and 3	1.05	0.83	0.94	0.63
HEAN -18 compounds 2 and 5	1.2	0.10	1.23	0.77
HEAN 1 crude extract	1.05	0.83	0.94	0.76
HEAN 19b compound 4	1.38	0.27	1.12	0.69
Diminazene	5.70	0.19	6.96	0.014
Suramin	0.62	0.50	0.87	0.74

RF, resistance factor, being the ratio of the EC50 values of the resistant and control strains. *p* value was obtained using unpaired Student’s test between the EC50 values of the resistant line and control, obtained in parallel (*n* = 3).

The PAN-76 and HEAN-1 mixtures and compound **4** were tested against HEK cells to determine whether their toxicity is selective to the parasites, or general. While the mixtures showed highest selectivity to *T. evansi* (Table 4), compound 4 is selective to *T. brucei, T. evansi*, and *L. mexicana* (SI = 21.2 – 33.8). Although compound **4** in purified form is more toxic to HEK cells than the crude fraction from which it was isolated, it remains low at ∼30 µM. Thus, compound **4** shows some promise as an antiprotozoal compound, especially against *Leishmania* spp. and will need to be further investigated.

**TABLE 4:** Toxicity of mixtures and compound 4 to HEK cells.

**Compound/mixture**	**EC**_**50**_ **for HEK cells (*n* = 4)**	**Selectivity index (SI)**				
		**SI** ***T. brucei* s427**	**SI** ***T. congolense* IL3000**	**SI** ***T. equiperdum***	**SI** ***T. evansi***	**SI** ***L. mexicana***
PAN-76 compound 1 and 3 (µg/ml)	56.8 ± 2.7	5.65	1.29	7.92	13.3	1.67
HEAN-1 crude (µg/ml)	75.5 ± 6.6	13.6	2.11	10.4	13.9	7.45
HEAN -19b compound 4 (µg/ml)	9.39 ± 1.37 (29.5 µM)	21.1	2.53	6.75	28.1	33.8
PAO (µM)	2.8 ± 0.08	—	—	—	—	—

**FIGURE 3:**
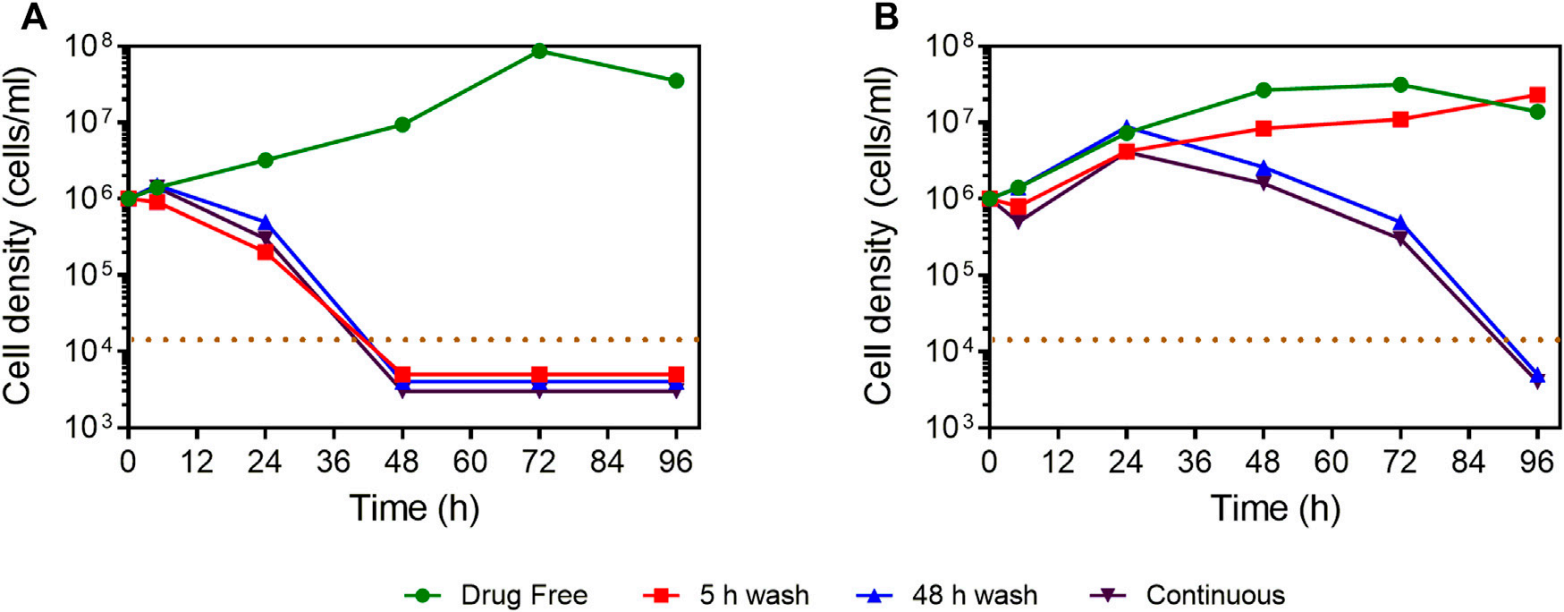
Manual cell count of *L. mexicana* cultures grown in the presence or absence of compound **4** and pentamidine at 5 × EC_50_, with or without wash after 5 or 48 h of incubation. **(A)** Compound **4**. **(B)** Pentamidine. The dotted brown line indicates the detection limit, being 10^4^ cells/ml. For convenience, where no cells were observed in the counting chamber, the values of 5,000, 4,000, or 3,000 cells/ml were entered to facilitate a graphical representation.

The selective antiparasitic activity of compound **4** prompted the investigation of its effect on growth of *L. mexicana* promastigotes *in vitro*. At a concentration of 5 × EC_50_, the compound irreversibly inhibited the growth of *L. mexicana* even after a short exposure of 5 h (Figure 3). In addition, the compound showed faster onset of action and killing than the control drug, pentamidine.

## Concluding Remarks

Due to the unprofitable market for kinetoplastid agents, new developments are unlikely to emerge through the regular discovery process of the pharmaceutical industry, especially for veterinary applications. Alternative local solutions are potentially available in the form of medicinal plants, a practice that is ongoing but requires scientific validation in order to delineate which extracts or isolated compounds from which plants work reliably against which pathogens. Here, we find that crude extracts of *Acacia nilotica* yielded two new diterpenes that had limited anti-kinetoplastid activity and one oxacassadiene, compound **4**, isolated from a fraction of the extract which displayed promising activity against several *Trypanosoma* species and *Leishmania mexicana*. Production of this compound from the widely available plant should now be scaled up for tests with further *Leishmania* species, intracellular amastigotes, and *in vivo* studies with relevant disease models.

## Data Availability

The datasets presented in this study can be found in online repositories. The names of the repository/repositories and accession number(s) can be found below: Figshare DOI: 10.6084/m9.figshare.13547816.
