# Supplementary material for: Two New Antiprotozoal Diterpenes From the Roots of Acacia nilotica
Source: Front Chem. 2021 Apr 21;9:624741. doi: 10.3389/fchem.2021.624741 (PMC8097170; doi:10.3389/fchem.2021.624741)
Supplement: Supplementary file 1 [file datasheet1.pdf]

# Supporting Information

**Table of Contents** **Page**


**Figure S1:** HR-EIMS spectrum of compound **1** [M-H]$^-$ ion at *m*/*z* = 319.2229 2

**Figure S2:** $^1$H-NMR (500 MHz, $CDCl_3$) Spectrum of compound **1** 3

**Figure S3:** $^1$H-NMR (500 MHz, $CDCl_3$) Spectrum of compound 1 (From $\delta_H$3.1 ppm to $\delta_H$ 4.7 ppm) 4

**Figure S4:** $^1$H-NMR (500 MHz, $CDCl_3$) Spectrum of compound 1 (From $\delta_H$6.6 ppm to $\delta_H$ 7.0 ppm) 5

**Figure S5:** $^1$H-NMR (500 MHz, $CDCl_3$) Spectrum of compound 1 (From $\delta_H$0.69 ppm to $\delta_H$ 0.87 ppm) 6

**Figure S6:** DEPTq135 (100 MHz, $CDCl_3$) Spectrum of compound **1** (From $\delta_C$ 50 ppm to $\delta_C$ 200 ppm ) 7

**Figure S7:** DEPTq135 (100 MHz, $CDCl_3$) Spectrum of compound **1** (From $\delta_C$ 11 ppm to $\delta_C$ 47 ppm) 8

**Figure S8:** HMBC Spectrum of compound **1**

**Figure S9:** HMBC Spectrum of compound **1** (From $\delta_H$ 3.50 ppm to $\delta_H$ 0.70 ppm)

HMBC Spectrum of compound **1** (From $\delta_C$ 15 ppm to $\delta_C$ 55 ppm ) 10

**Figure S10:** NOESY Spectrum of compound 1 11

**Figure S11**: COSY Spectrum of compound **1** 12

**Figure S12:** HR-EIMS spectrum LC-MS spectrum of compound 2 at m/z = 318.2195 13

**Figure S13:** $^1$H NMR spectrum of compound 2 14

**Figure S14:** $^1$H NMR spectrum of compound 2 (annotated) 15

**Figure S15:** $^{13}$C DEPTq-135 spectrum of compound 2 **16**

**Figure S16:** HMBC Spectrum of compound 2 17

**Figure S17:** COSY Spectrum of compound 2 18

**Figure S18:** HSQC Spectrum of compound 2

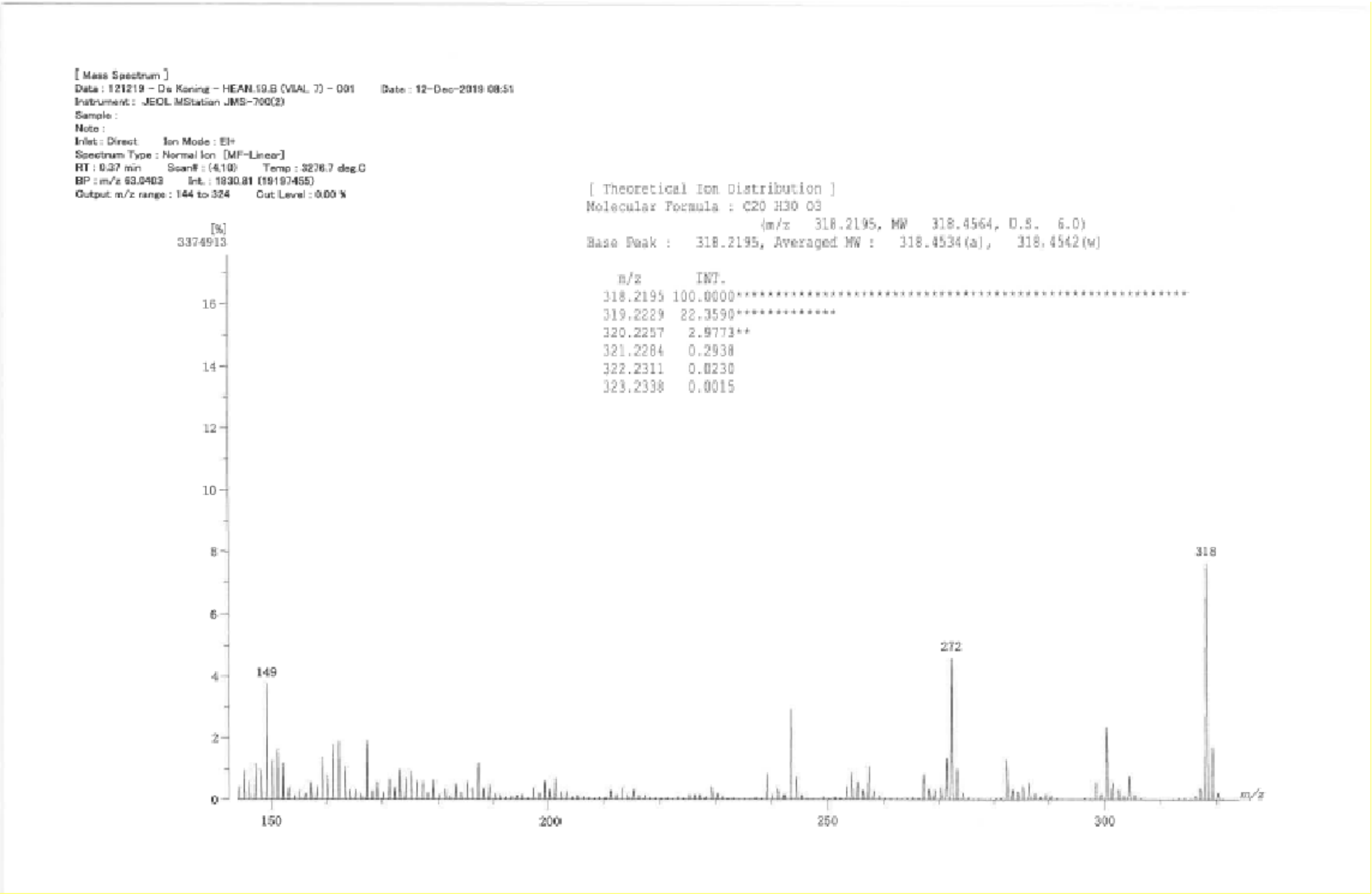


**Figure S1:** HR-EIMS spectrum of compound **1** [M-H]$^-$ ion at *m*/*z* = 319.2229

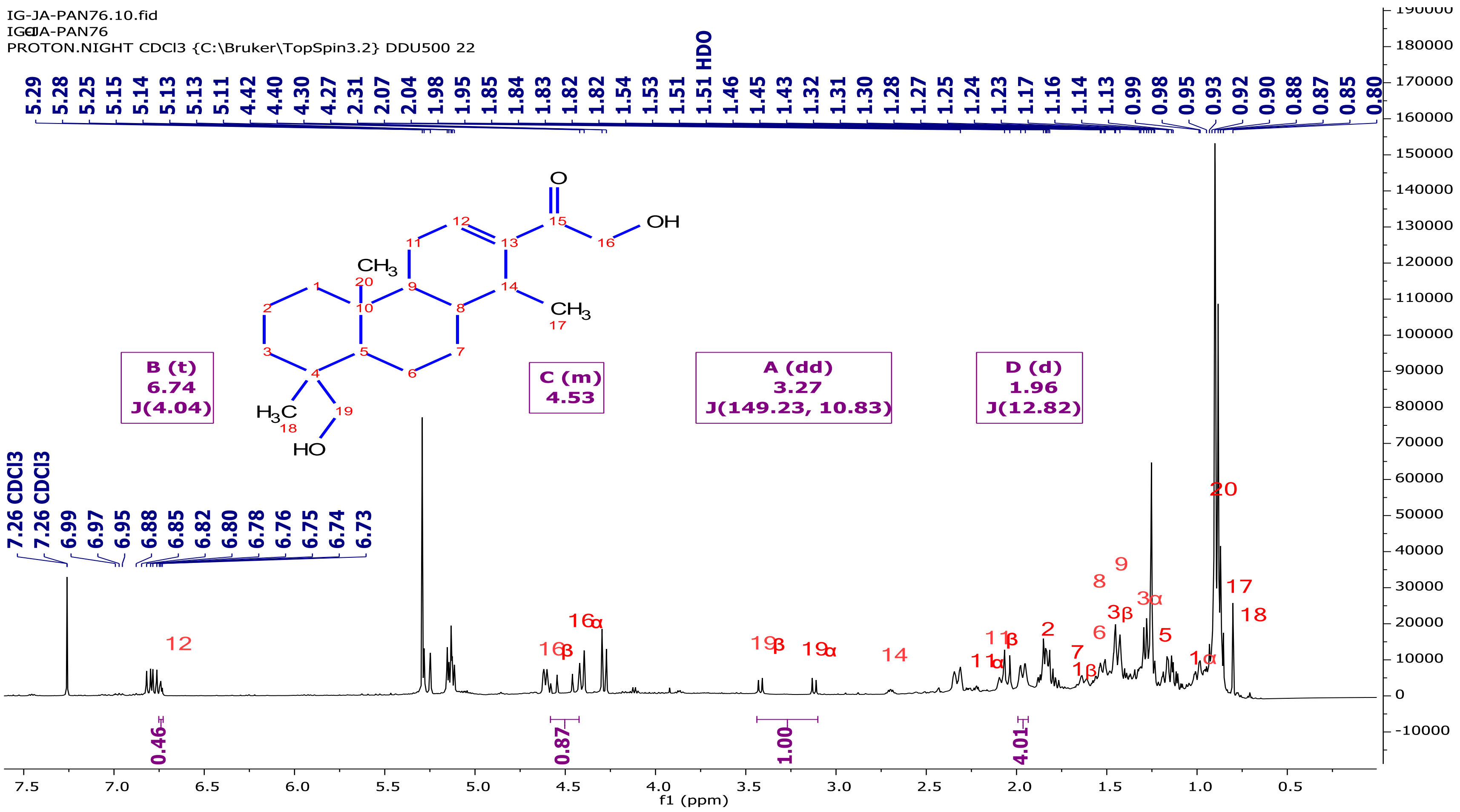


**Figure S2:** $^{1}$H-NMR (400 MHz, $CDCl_3$) Spectrum of compound **1**

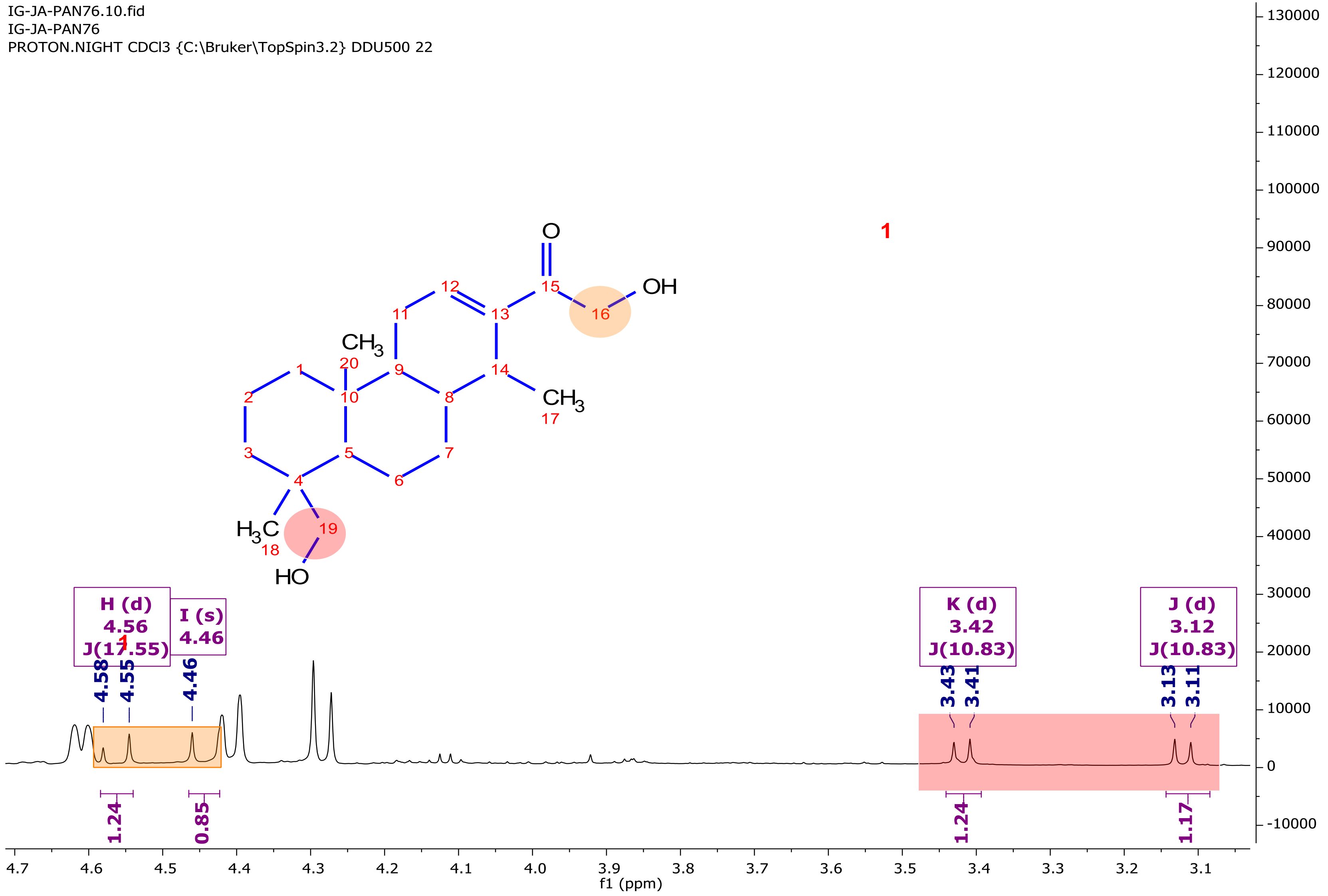


**Figure S3:** $^{1}$H-NMR (400 MHz, $CDCl_3$) Spectrum of compound 1 (From $\delta_H$3.1 ppm to $\delta_H$ 4.7 ppm)

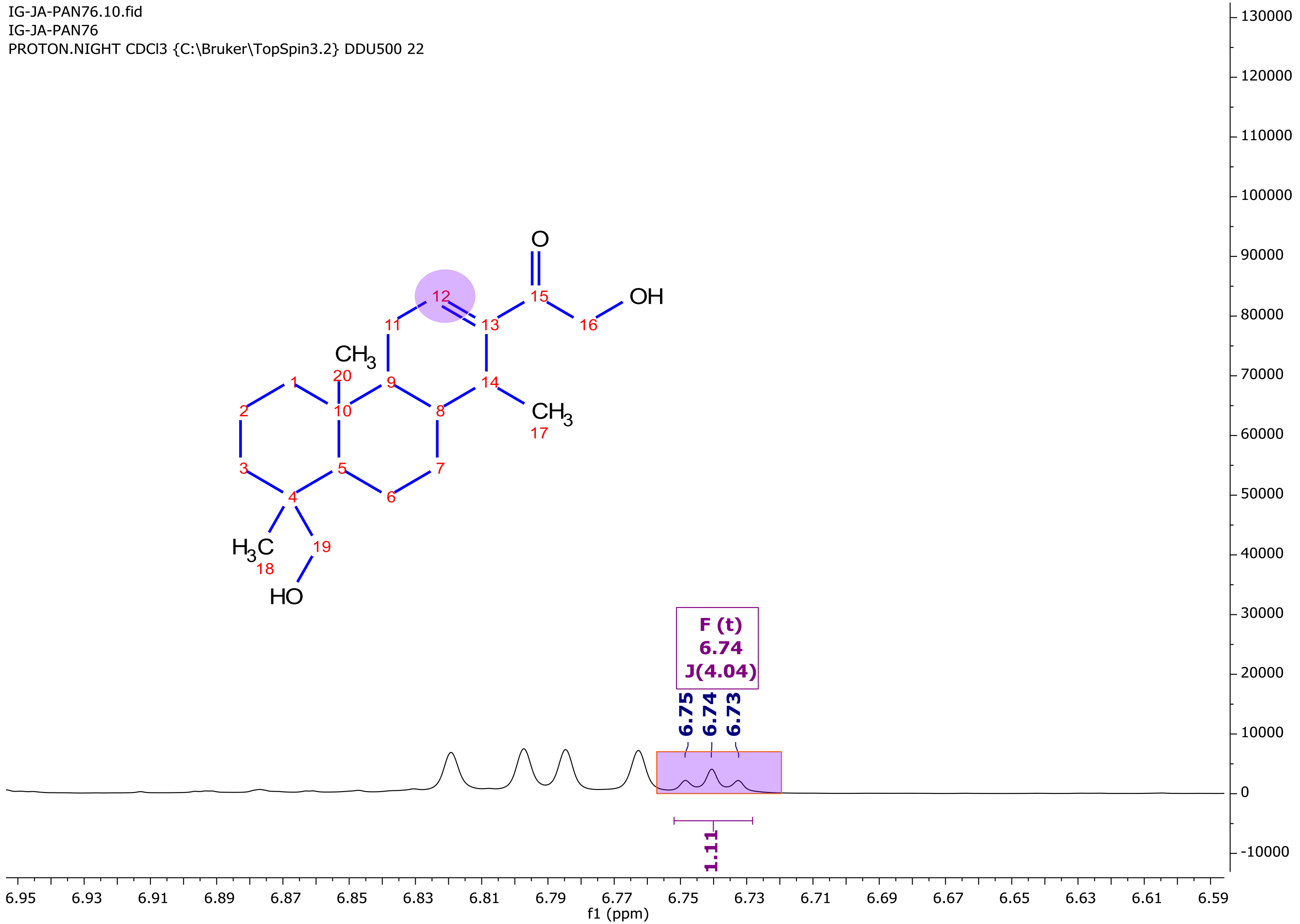


**Figure S4:** $^1$H-NMR (400 MHz, $CDCl_3$) Spectrum of compound 1 (From $\delta_H$6.6 ppm to $\delta_H$ 7.0 ppm)

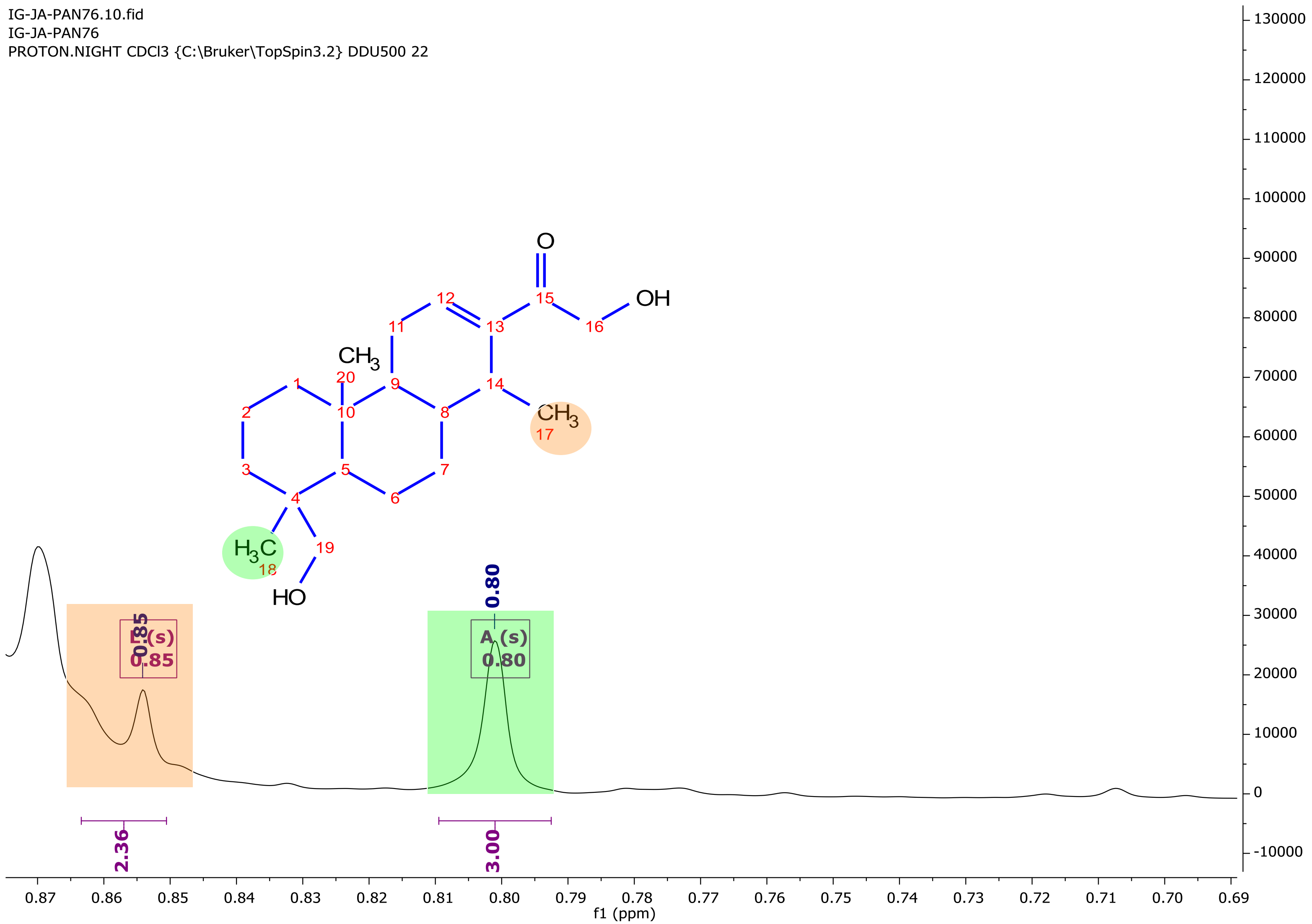


**Figure S5:** $^{1}$H-NMR (400 MHz, $CDCl_3$) Spectrum of compound 1 (From $\delta_H$0.69 ppm to $\delta_H$ 0.87 ppm)

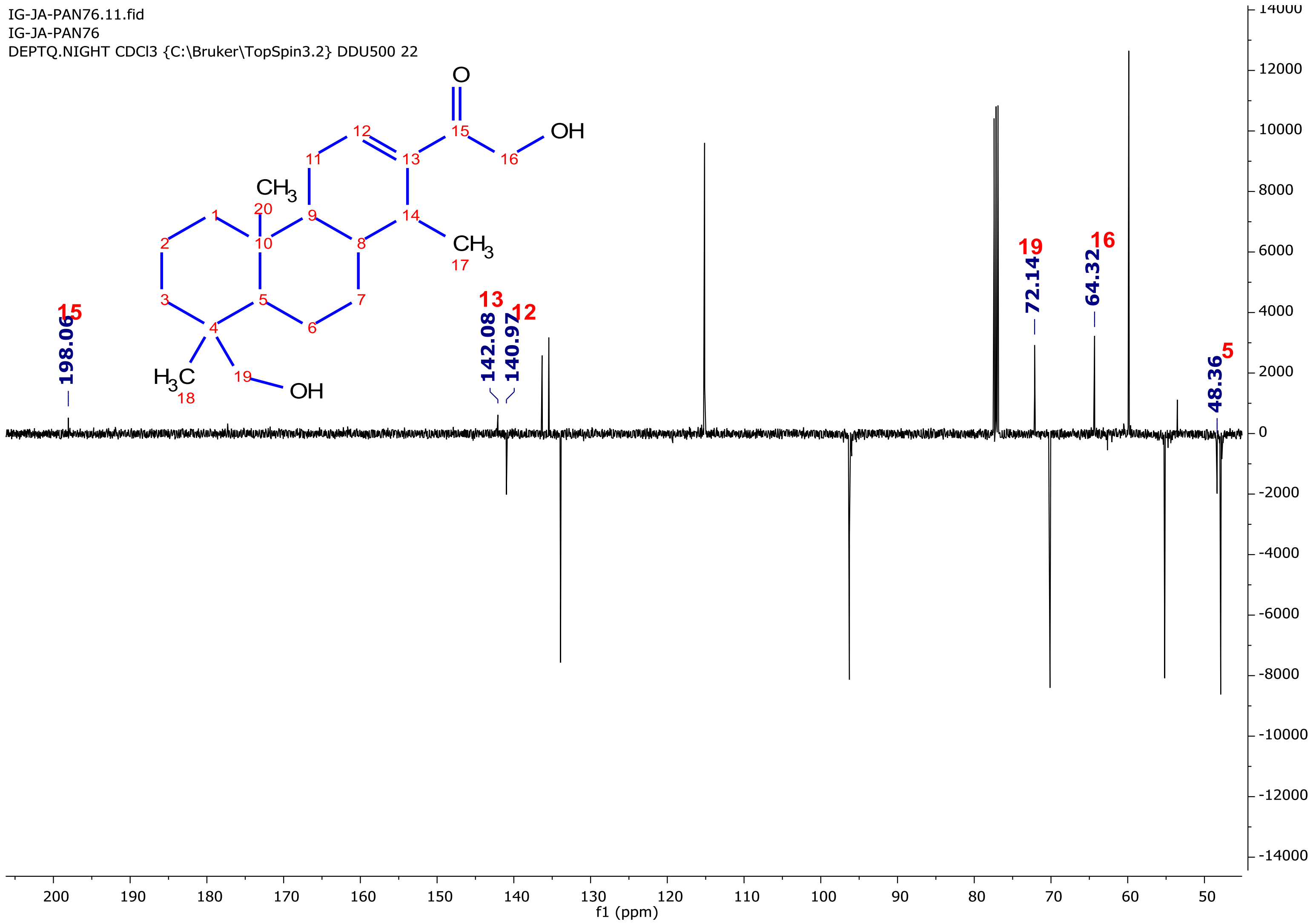


**Figure S6**: DEPTq135 (100 MHz, $CDCl_3$) Spectrum of compound 1 (From $\delta_C$ 50 ppm to $\delta_C$ 200 ppm)

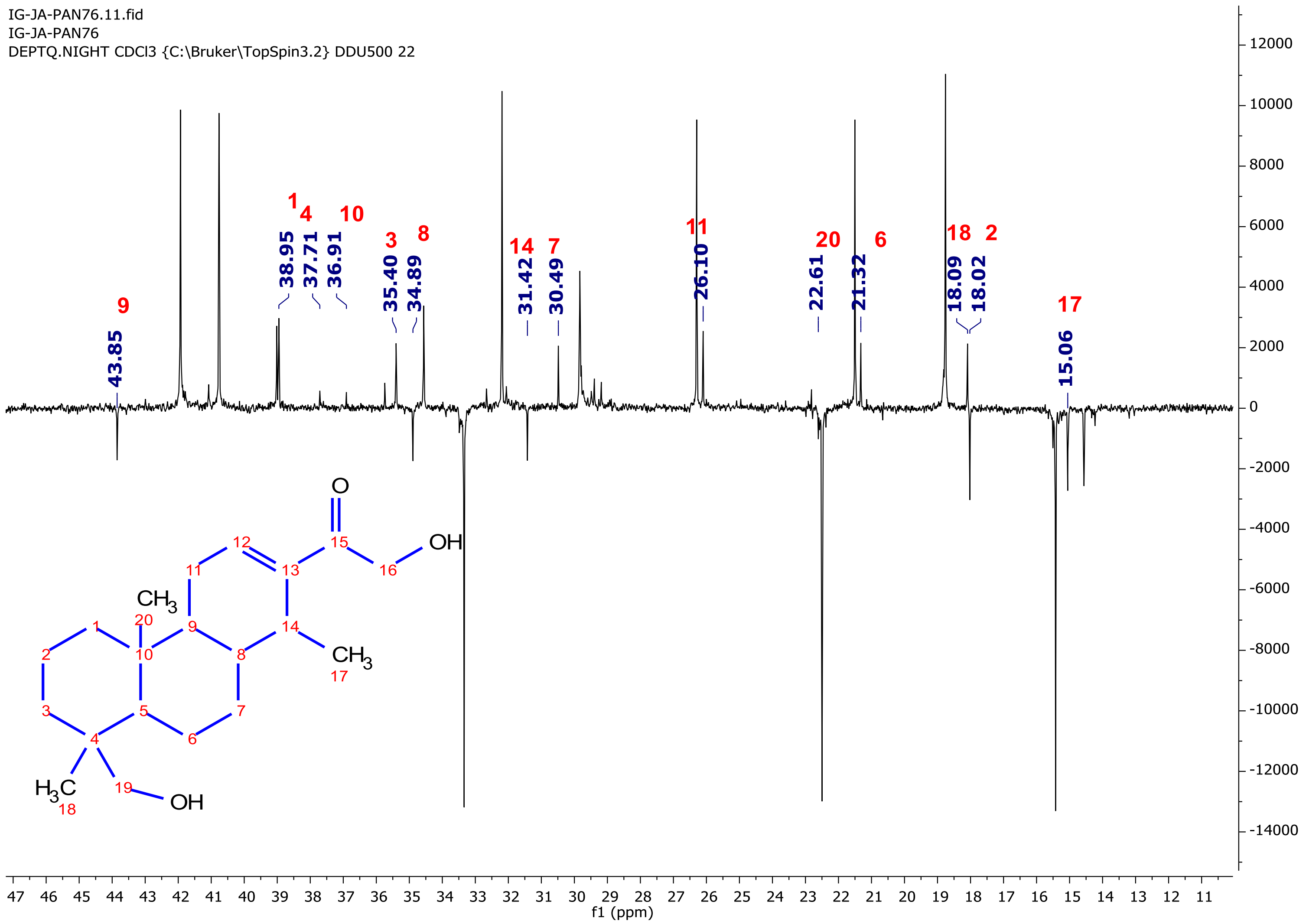


**Figure S7**: DEPTq135 (100 MHz, $CDCl_3$) Spectrum of compound 1 (From $\delta_C$ 11 ppm to $\delta_C$ 47 ppm)

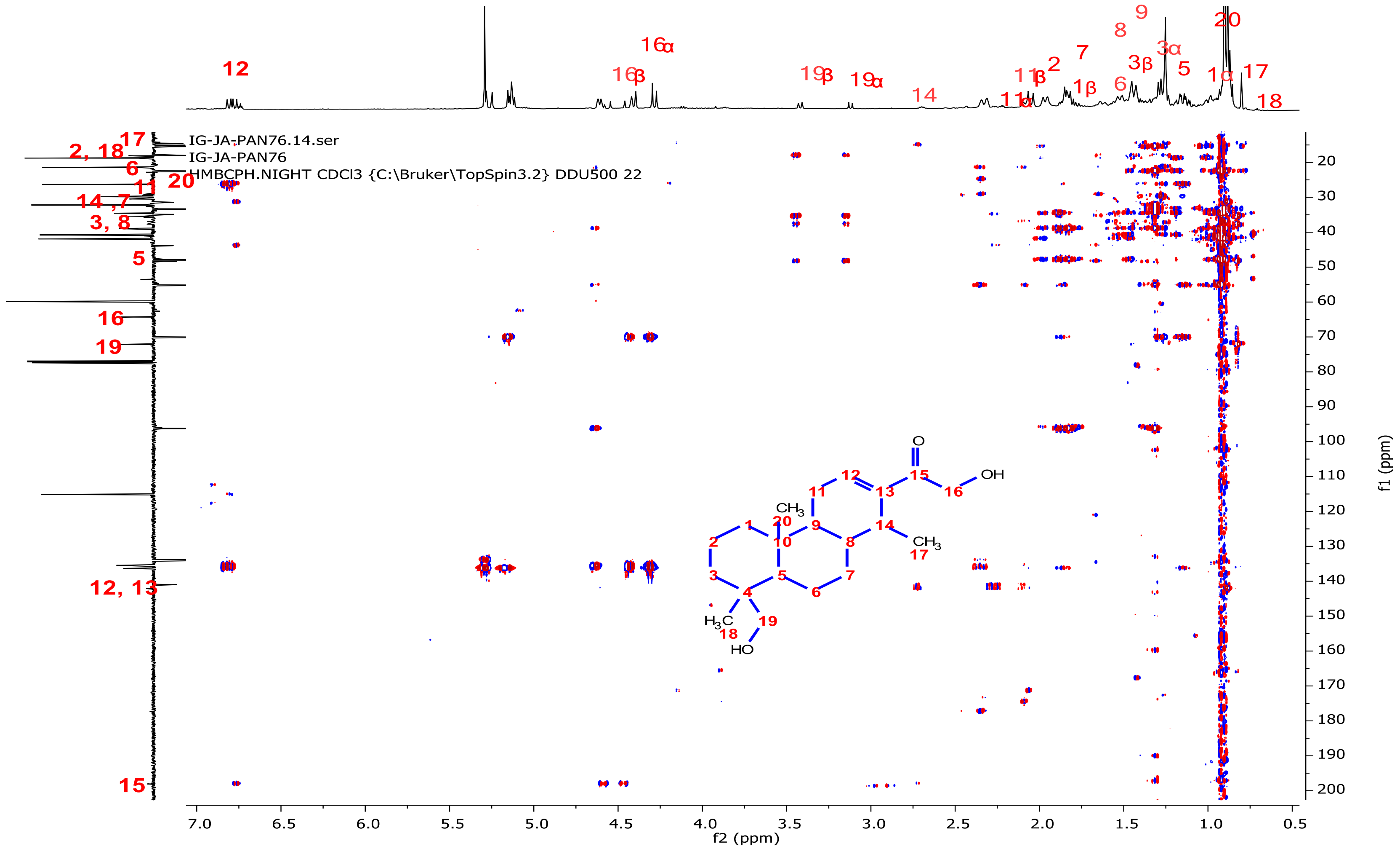


**Figure S8**: HMBC Spectrum of compound 1

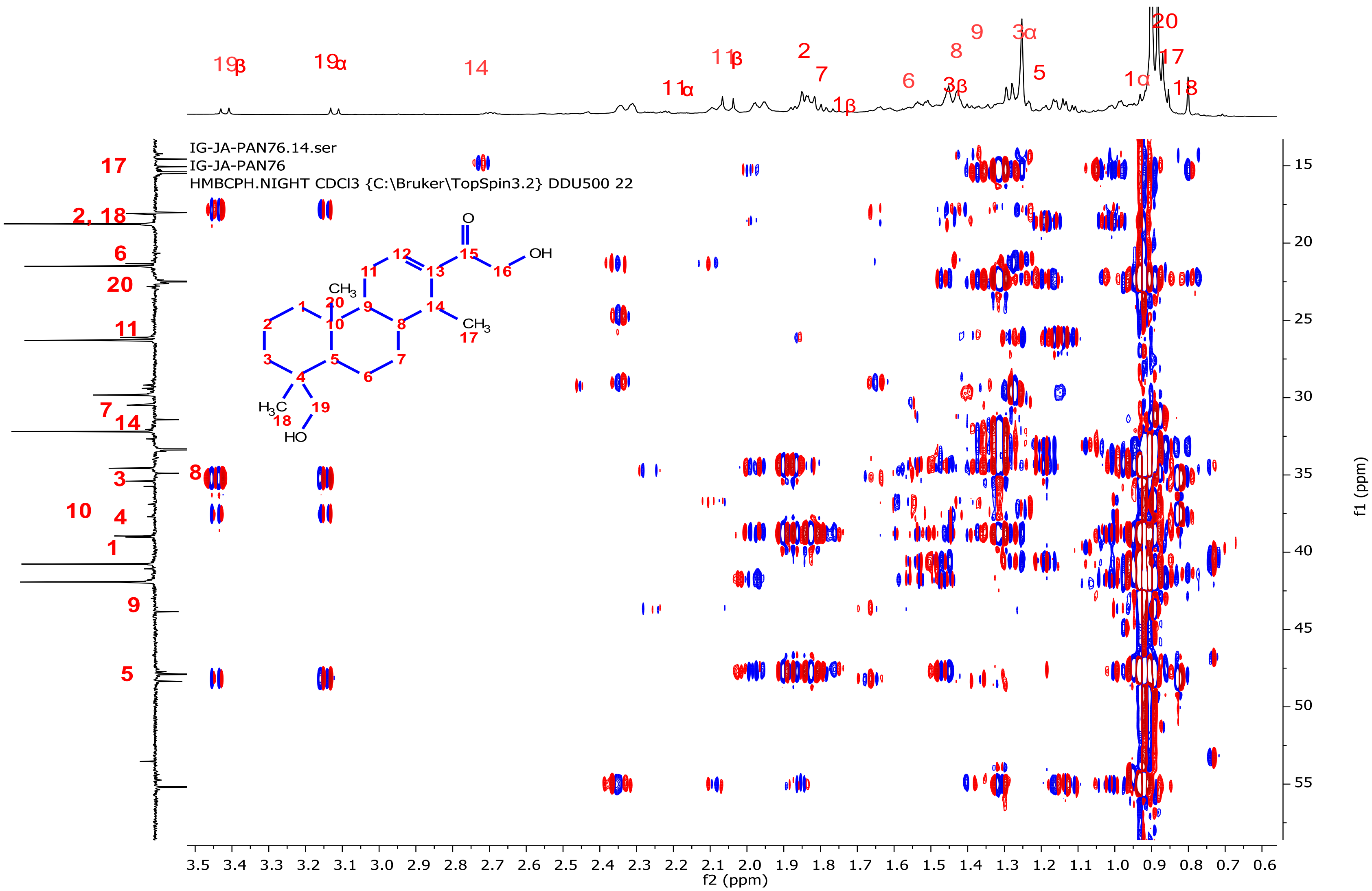


**Figure S9**: HMBC Spectrum of compound 1 (From $\delta_H$ 3.50 ppm to $\delta H$ 0.70 ppm)/($\delta_C$ 15 ppm to $\delta_C$ 55 ppm )

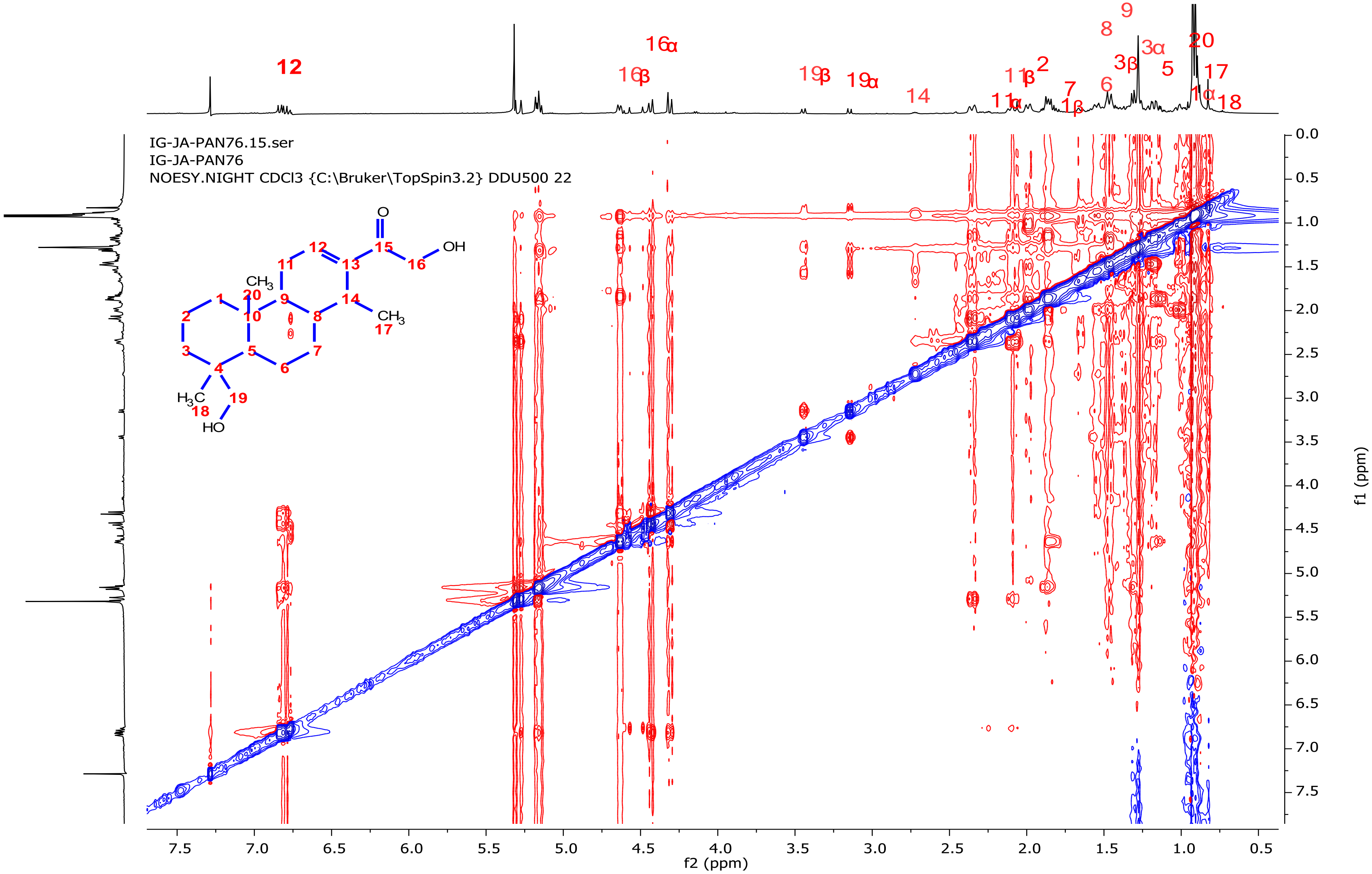


**Figure S10**: NOESY Spectrum of compound 1

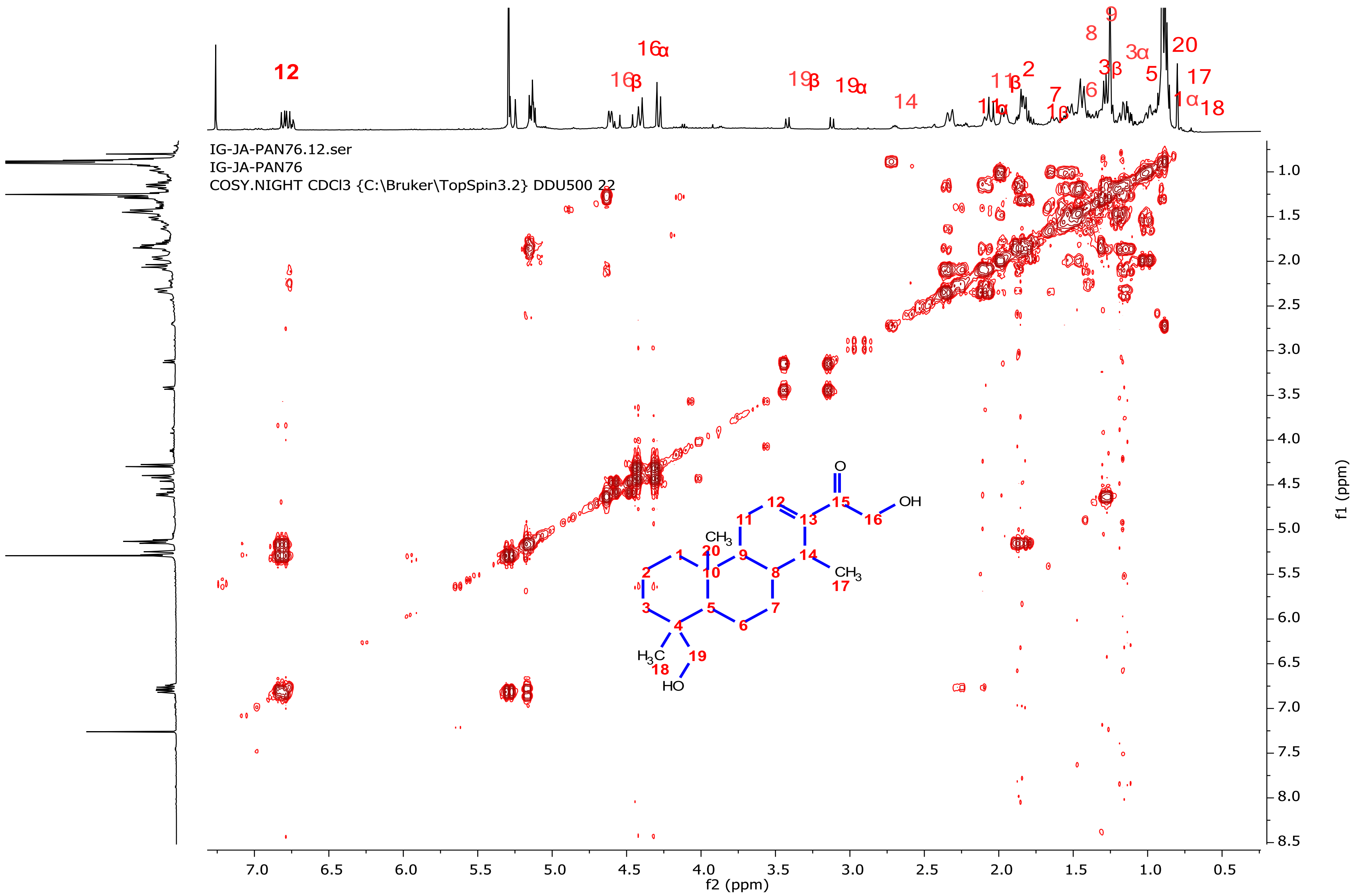


**Figure S11**: COSY Spectrum of compound 1

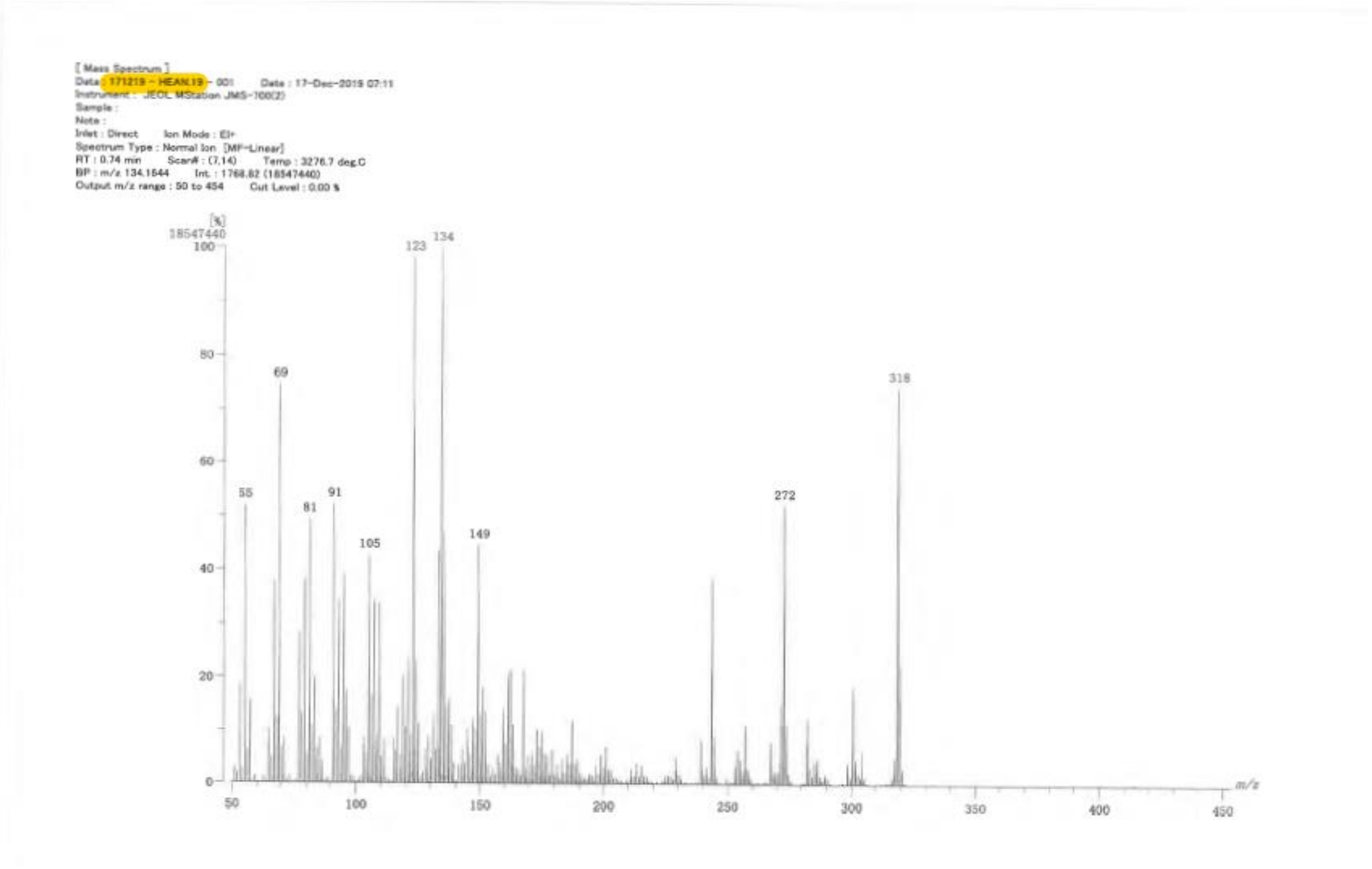


**Figure S12**: HR-EIMS spectrum of compound **2** at *m*/*z* = 318.2195

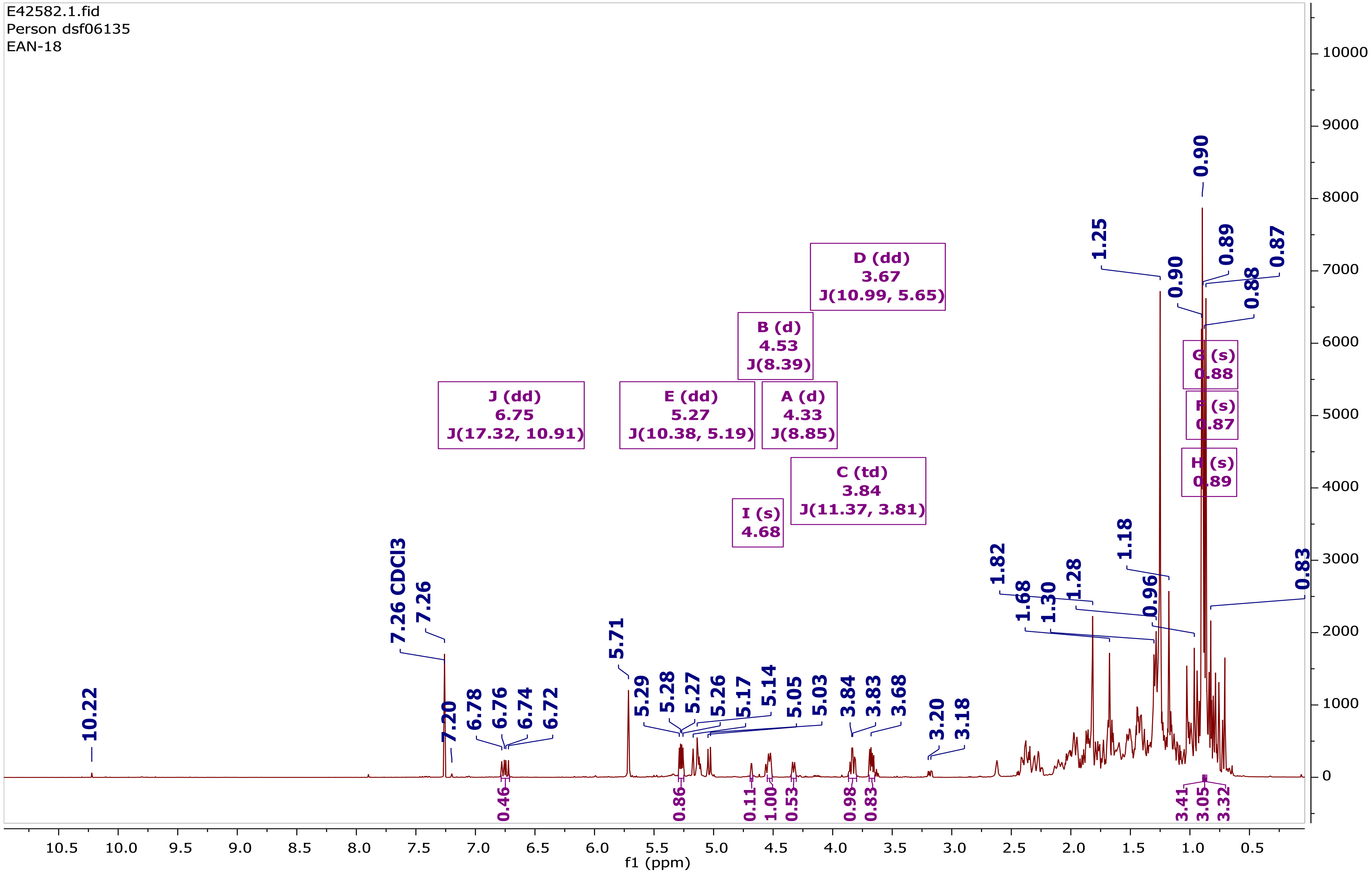


**Figure S13**: $^{1}$H NMR Spectrum of compound 2

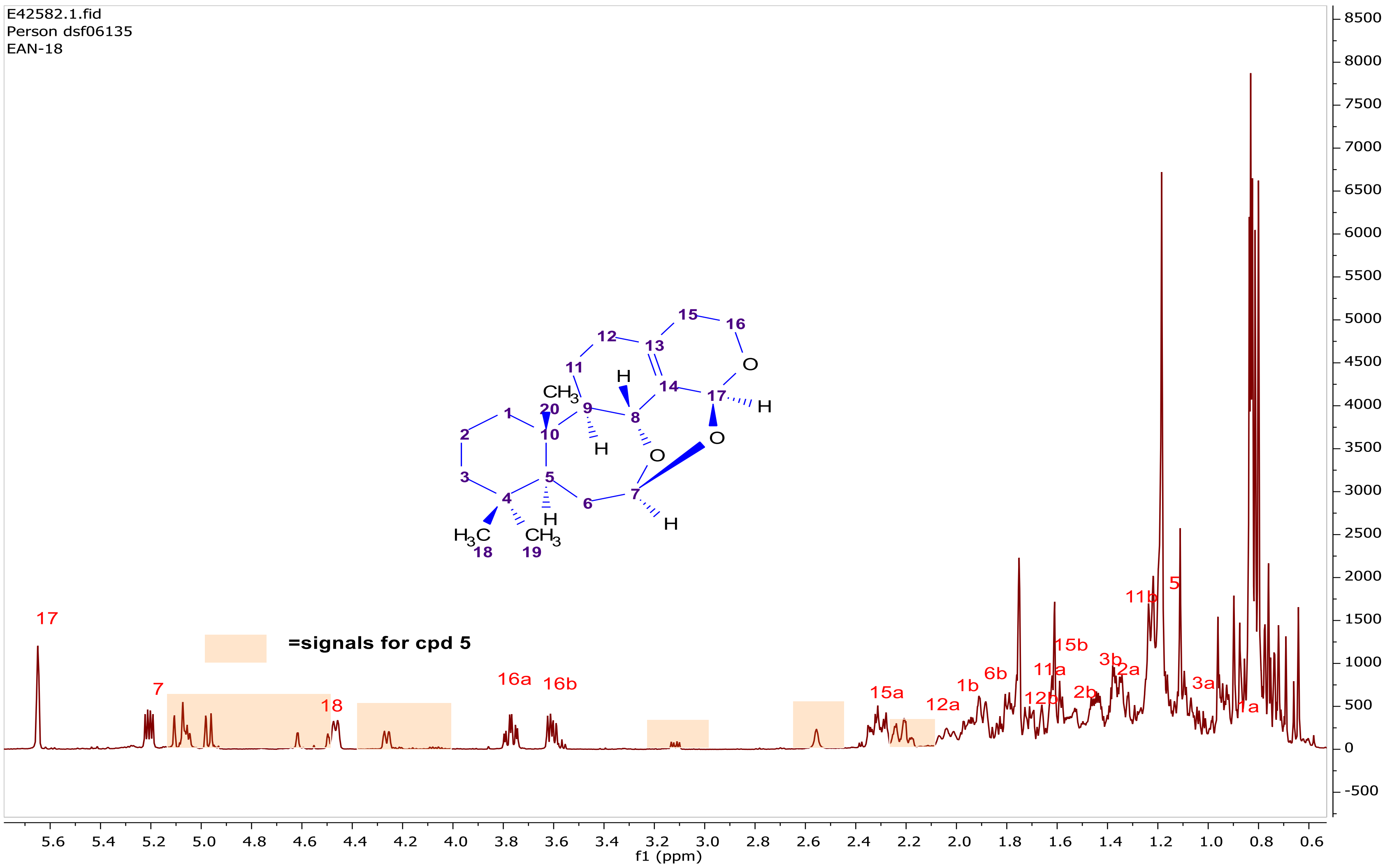


**Figure S14**: $^{1}$H NMR Spectrum of compound 2 (annotated).

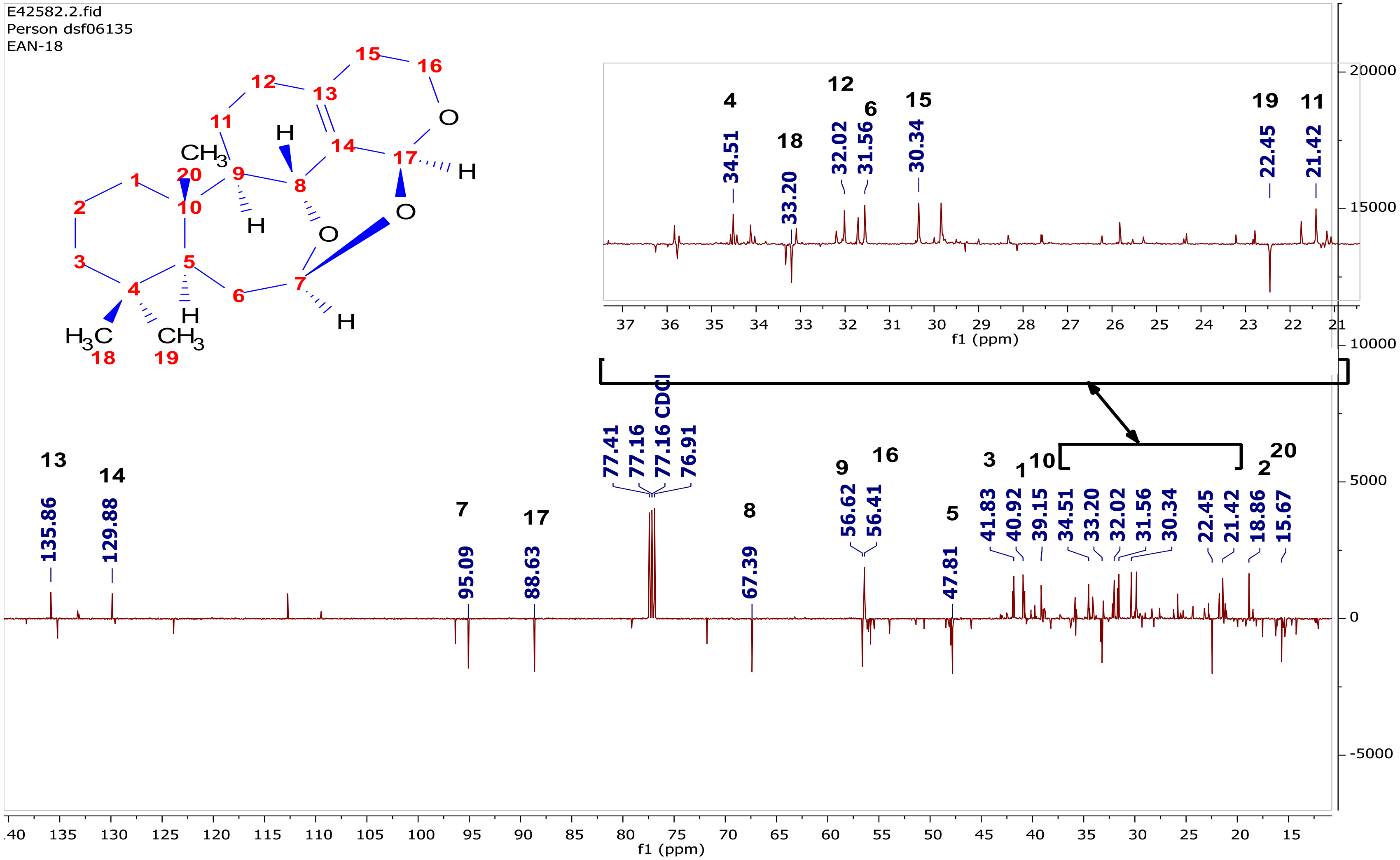


**Figure S15**: $^{13}$C DEPTq-135 Spectrum of compound 2

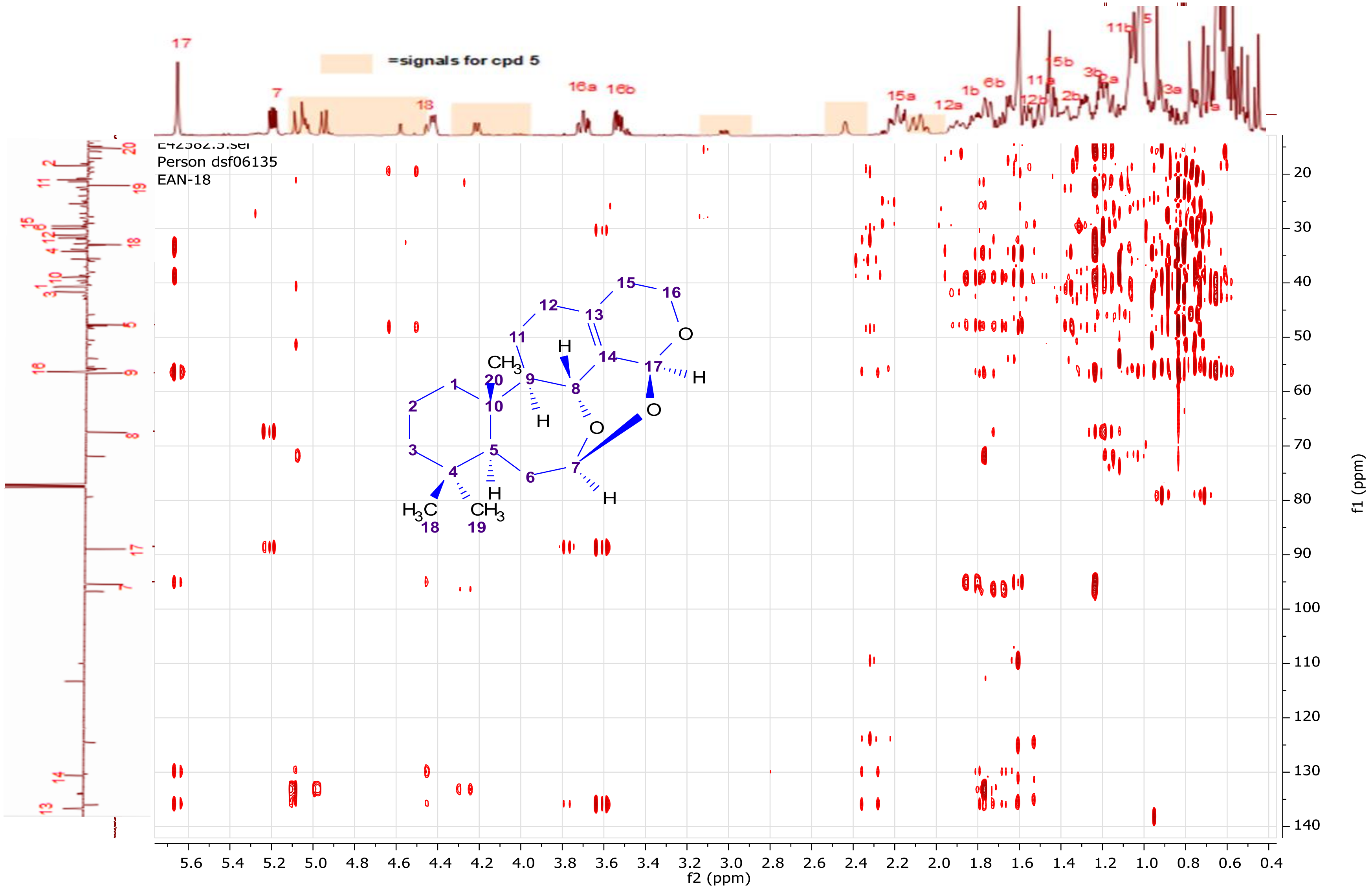


**Figure S16**: HMBC Spectrum of compound 2

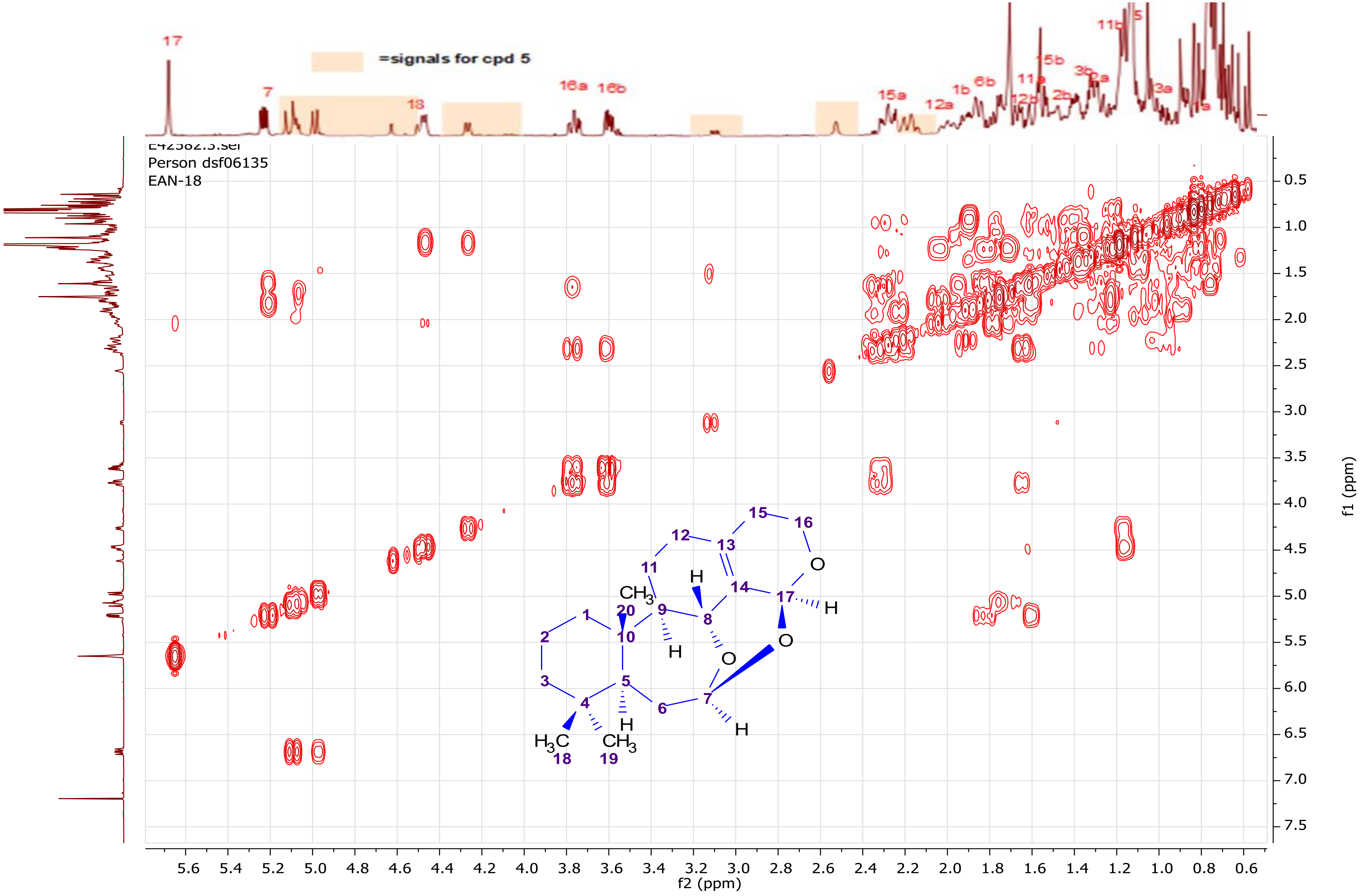


**Figure S17**: COSY Spectrum of compound 2

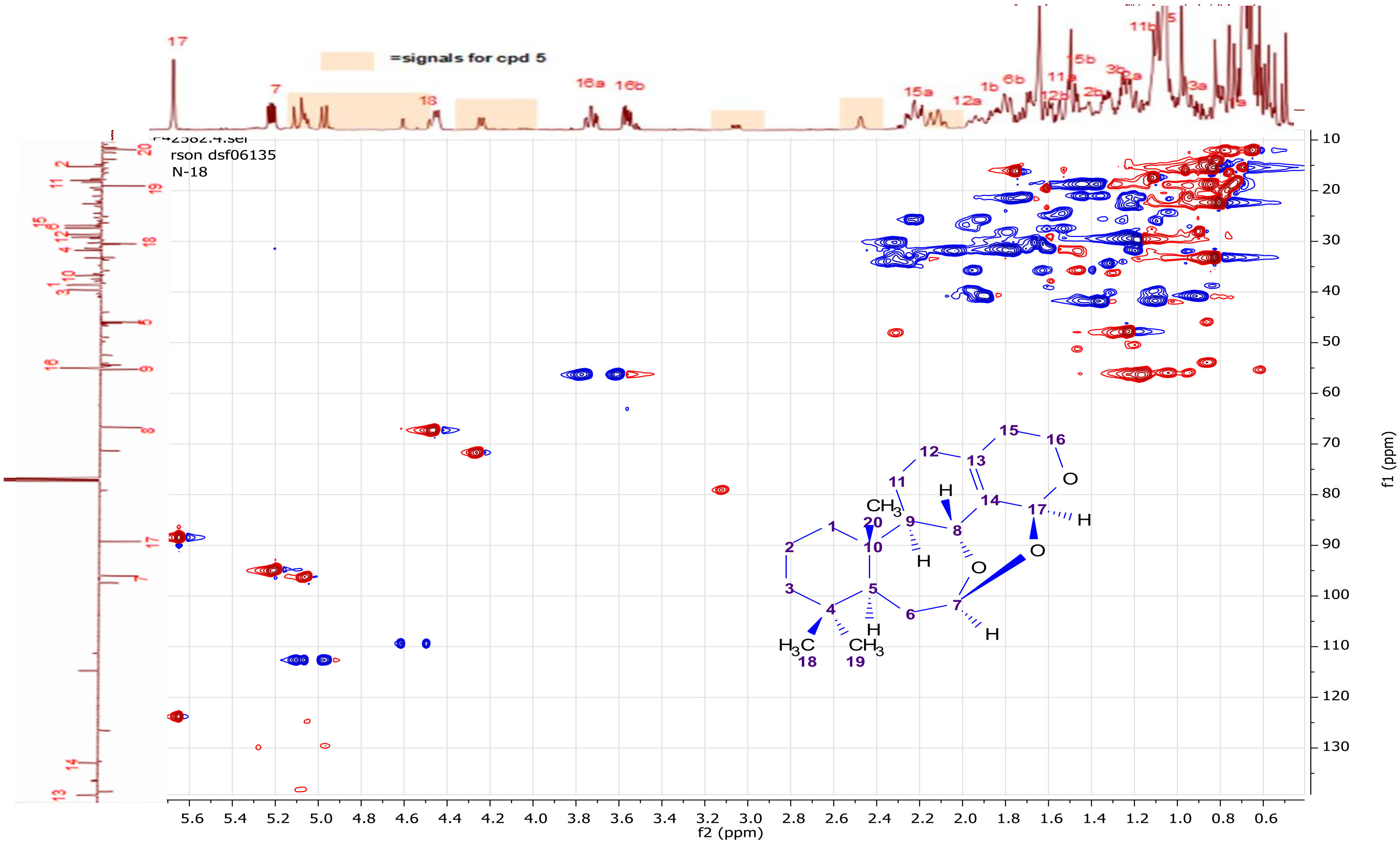


**Figure S18**: HSQC Spectrum of compound 2
